# The alternative splicing factor Nova2 regulates vascular development and lumen formation

**DOI:** 10.1038/ncomms9479

**Published:** 2015-10-08

**Authors:** Costanza Giampietro, Gianluca Deflorian, Stefania Gallo, Anna Di Matteo, Davide Pradella, Serena Bonomi, Elisa Belloni, Daniel Nyqvist, Valeria Quaranta, Stefano Confalonieri, Giovanni Bertalot, Fabrizio Orsenigo, Federica Pisati, Elisabetta Ferrero, Giuseppe Biamonti, Evelien Fredrickx, Carla Taveggia, Chris D. R. Wyatt, Manuel Irimia, Pier Paolo Di Fiore, Benjamin J. Blencowe, Elisabetta Dejana, Claudia Ghigna

**Affiliations:** 1 FIRC Institute of Molecular Oncology, Via Adamello 16, Milan 20139, Italy; 2 Department of Biosciences, Milan University, Via Celoria 26, Milan 20133, Italy; 3 Istituto di Genetica Molecolare, Consiglio Nazionale delle Ricerche, via Abbiategrasso 207, Pavia 27100, Italy; 4 IUSS—Istituto Universitario di Studi Superiori, Piazza della Vittoria 15, Pavia 27100, Italy; 5 Dipartimento di Biologia e Biotecnologie, `Lazzaro Spallanzani' - Università degli Studi di Pavia, via Ferrata 9, Pavia 27100, Italy; 6 Division of Vascular Biology, Department of Medical Biochemistry and Biophysics, Karolinska Institutet, Stockholm 171 77, Sweden; 7 Dipartimento di Oncologia Sperimentale, Istituto Europeo di Oncologia, Milan 20141, Italy; 8 Department of Oncology, San Raffaele Scientific Institute, via Olgettina 58, Milan 20132, Italy; 9 Division of Neuroscience and INSPE at San Raffaele Scientific Institute, via Olgettina 58, Milan 20132, Italy; 10 EMBL/CRG Research Unit in Systems Biology, Centre for Genomic Regulation (CRG), Barcelona 08003, Spain; 11 Universitat Pompeu Fabra (UPF), Barcelona 08003, Spain; 12 Dipartimento di Scienze della Salute, University of Milan, Milan 20122, Italy; 13 Donnelly Centre for Cellular and Biomolecular Research, University of Toronto, 160 College Street, Toronto, Ontario, Canada M5S 3E1; 14 Rudbeck Laboratory and Science for Life Laboratory, Department of Immunology, Genetics and Pathology, Uppsala University, Dag Hammarksjöldsv 20, Uppsala 751 85, Sweden

## Abstract

Vascular lumen formation is a fundamental step during angiogenesis; yet, the molecular mechanisms underlying this process are poorly understood. Recent studies have shown that neural and vascular systems share common anatomical, functional and molecular similarities. Here we show that the organization of endothelial lumen is controlled at the post-transcriptional level by the alternative splicing (AS) regulator Nova2, which was previously considered to be neural cell-specific. Nova2 is expressed during angiogenesis and its depletion disrupts vascular lumen formation *in vivo*. Similarly, Nova2 depletion in cultured endothelial cells (ECs) impairs the apical distribution and the downstream signalling of the Par polarity complex, resulting in altered EC polarity, a process required for vascular lumen formation. These defects are linked to AS changes of Nova2 target exons affecting the Par complex and its regulators. Collectively, our results reveal that Nova2 functions as an AS regulator in angiogenesis and is a novel member of the ‘angioneurins' family.

## Abstract (web summary)

The alternative splicing factor Nova2 is best known for its pivotal function in the brain. Giampietro *et al*. reveal an important role for Nova2 in the regulation of alternative splicing of transcripts in the vascular endothelium that are crucial for the maintenance of endothelial cell polarity and vessel lumen formation in zebrafish.

## Graphical abstract

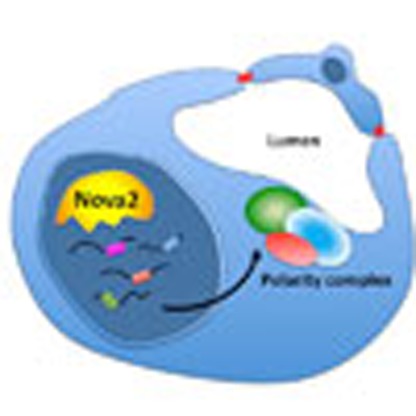

In adulthood most blood vessels remain quiescent; however, in conditions of active physiological tissue growth, such as during embryogenesis or tissue repair, endothelial cells (ECs) migrate and proliferate to form new vessels. This process, known as angiogenesis, is also critical for the pathogenesis of several disorders and to support cancer development and progression^1^. Although angiogenesis does not initiate malignancy, it promotes tumour growth by allowing oxygen and nutrients to reach proliferating cancer cells. Targeting angiogenesis represents a particularly promising anticancer therapeutic approach, and several strategies have been attempted so far^2^.

Recent studies have highlighted significant anatomic, structural and molecular similarities between the vascular and the nervous systems^3^. Both systems possess specialized structures—tip cells at the forefront of endothelial sprouts and axonal growth cones—that, through filopodial extensions, probe the environment for guidance cues. Molecules regulating these processes have been termed ‘angioneurins'^3^. The prototypic angioneurin is the vascular endothelial growth factor (VEGF), which was originally discovered as a key angiogenic factor, but subsequently shown to be important also in the development of the nervous system^4^. Since blood vessels and nerves are functionally interdependent, the malfunctioning of this ‘neurovascular link' can lead to several vascular and neuronal disorders^3^.

Until now, the many molecular pathways regulating vascular development and angiogenesis have been suggested to act primarily through the regulation of transcription. However, recent studies indicate that post-transcriptional and epigenetic programmes cooperate to confer tissue-specific vascular properties.

Alternative splicing (AS) is a molecular process that generates multiple, distinct mature mRNAs from a primary transcript (pre-mRNA), leading to the production of protein isoforms with different structural and functional properties. Since more than 90% of human multiexonic genes undergo AS^5^,^6^, this process represents a major mechanism underlying the expansion of the proteome from a limited repertoire of genes^7^,^8^. AS and transcription predominantly regulate different subsets of genes to generate the molecular and cellular complexity of different cell and tissue types^9^,^10^,^11^. AS thus provides a versatile, additional layer of regulation to both establish and maintain fundamental properties of different cell and tissue types. Despite the importance of AS, the functional roles of the vast majority of AS events is not well understood.

While there are several examples of splicing variants with a role in angiogenesis^12^,^13^,^14^,^15^, the molecular mechanisms responsible for their production are still unknown. Here we describe a novel role for Nova2, previously described as neural cell-specific^16^, as a key AS regulator of angiogenesis. Both its expression and the levels of AS of its target exons are regulated during this process. Through gain- and loss-of-function approaches in ECs, we show that Nova2 regulates AS of factors belonging to the Par polarity complex and its regulators. Consequently, vascular lumen formation defects are developed in zebrafish on *nova2* morpholino-mediated knockdown or clustered regularly interspaced short palindromic repeat (CRISPR)-induced genetic mutation. Collectively, our results provide evidence that Nova2 is a new member of the ‘angioneurins' family, and further highlight an important biological role for post-transcriptional regulation of exon networks that contribute to both vascular and neuronal functions.

## Results

### Nova2 expression and function are regulated in ECs

To identify splicing regulatory factors (SRFs) involved in endothelial growth and quiescence, we studied ECs under sparse and confluent conditions. By mining previously published Affymetrix gene expression data^17^ comparing mouse ECs grown at different densities, we identified *Nova2* as an SRF that is significantly upregulated in confluent versus sparse ECs (fold change=2.3; *P* value<0.05, Dunnett test). This result was surprising since Nova2 was considered previously to be neural cell-specific^16^.

Nova2 and its paralogue Nova1 are among the best-studied mammalian tissue-specific SRFs. Both proteins bind RNA through KH domains that recognize clusters of YCAY repeats within the pre-mRNA targets^16^. These factors, with indistinguishable biochemical properties but mutually exclusive expression within the central nervous system (CNS)^18^, regulate AS programmes involved in neuronal development and synapse activity^16^.

**Figure 1:**
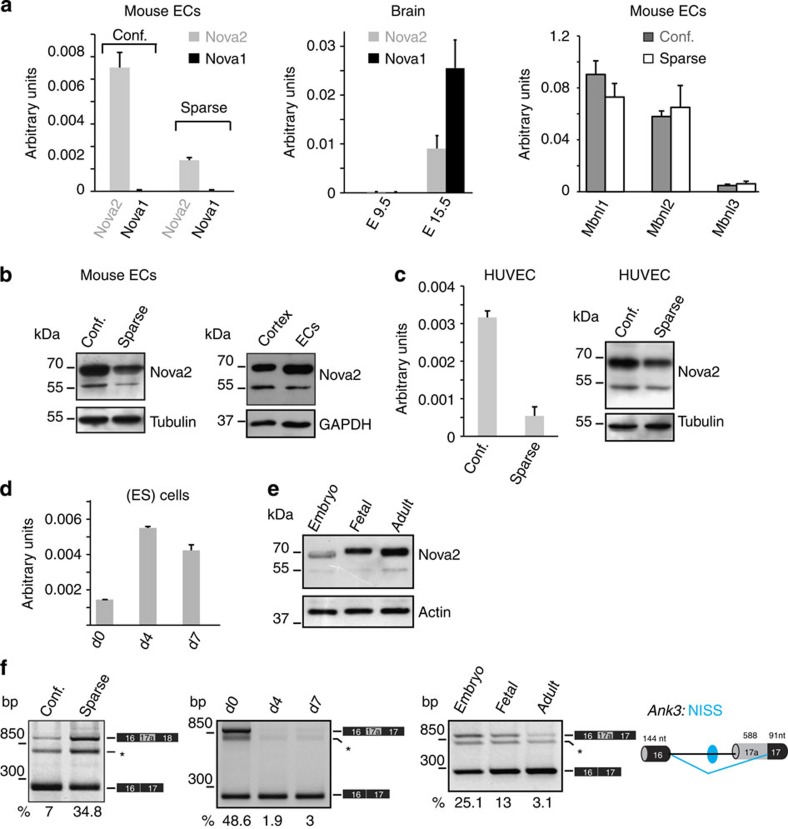
Nova2 expression levels and AS of its target are regulated in ECs. (**a**) RT–qPCR analysis of *Nova2* and *Nova1* mRNA expression levels in mouse ECs grown as confluent or sparse (left), in E9.5 and E15.5 mouse whole brain (centre), and RT–qPCR analysis of Muscleblind family members (*Mbnl1*, *Mbnl2* and *Mbnl3*) in mouse ECs grown at different densities (right). (**b**) Immunoblotting analysis of Nova2 levels in mouse confluent and sparse ECs (left panel; Tubulin as the loading control) and in confluent ECs and the mouse brain cortex (right panel; GAPDH as loading control). (**c**) Nova2 mRNA and protein expression levels in HUVECs grown at different densities. (**d**) RT–qPCR analysis of *Nova2* during endothelial differentiation of mouse ES cells at the indicated times. (**e**) Immunoblotting analysis of Nova2 in mouse EC lines derived from whole embryo, fetal heart and adult lung; Actin as the loading control. In all histograms, error bars indicate ±s.d. calculated from three independent experiments (*n*=3). (**f**) RT–PCR analysis of AS of a known Nova2 target (*Ankyrin3*/*Ank3*) in mouse ECs (confluent and sparse; left), during endothelial differentiation of mouse ES cells (centre) and in mouse ECs of different origins (right). The schematic representation of the mouse gene structure (AS exon in grey; constitutive exons in black), the YCAY cluster predicted to function as Nova2 silencer (blue dot) and the Nova2-regulated exon-skipping event (blue bars) are also shown. NISS, Nova intronic splicing silencer. The percentage of exon inclusion is shown. Asterisk indicates a nonspecific PCR product.

Validating the microarray results, we confirmed Nova2 upregulation in confluent versus sparse ECs using reverse transcription–quantitative PCR (RT–qPCR; Fig. 1a). By comparing *VE-cadherin*-null ECs (VEC-null) with the same cells reconstituted with *VE-cadherin* (VEC-positive)^17^ we also found that *Nova2* upregulation in confluent versus sparse ECs does not require VE-cadherin expression (Supplementary Fig. 1A). On the contrary, we found that *Nova1* is expressed to a negligible level in ECs, while both factors are expressed in E15.5 mouse whole brain that we used as positive control (Fig. 1a), consistent with recently published results^19^,^20^. Moreover, expression of the Muscleblind family of tissue-specific AS regulators (*Mbnl1*, *Mbnl2* and *Mbnl3*) was not modified by confluence in ECs (Fig. 1a). We further confirmed the upregulation of Nova2 in confluent ECs at the protein level by immunoblotting (Fig. 1b). Comparable results were obtained by using another EC line (adult ECs from the mouse lung) under sparse and confluent conditions (Supplementary Fig. 1B). Notably, as in the case of mouse cortex (Fig. 1b) and in human neuroblastoma SH-SY5Y cells (Supplementary Fig. 1C), in ECs the anti-Nova2-specific antibody recognized two immunoreactive bands at 50–55 and 70–80 kDa, as previously reported^18^. In agreement with available RNA sequencing (RNAseq) data (Supplementary Fig. 1D), we found that *Nova2* is also expressed in primary human umbilical vein endothelial cells (HUVECs) and that its levels decreased when HUVECs were grown as sparse (Fig. 1c). Moreover, *Nova2* expression increased during endothelial differentiation of mouse embryonic stem (ES) cells (Fig. 1d), or in adult ECs as compared with embryo or fetal ECs (Fig. 1e). Importantly, Nova2 expression correlated with AS changes of its known target *Ank3*: (i) in sparse versus confluent ECs, (ii) during endothelial differentiation of ES cells and (iii) in ECs of different origin (Fig. 1f) and AS of this target parallels that observed in brain of *Nova2*-null mice^21^. Taken together, these data suggest that Nova2 expression and function may play a role in vascular maturation.

**Figure 2:**
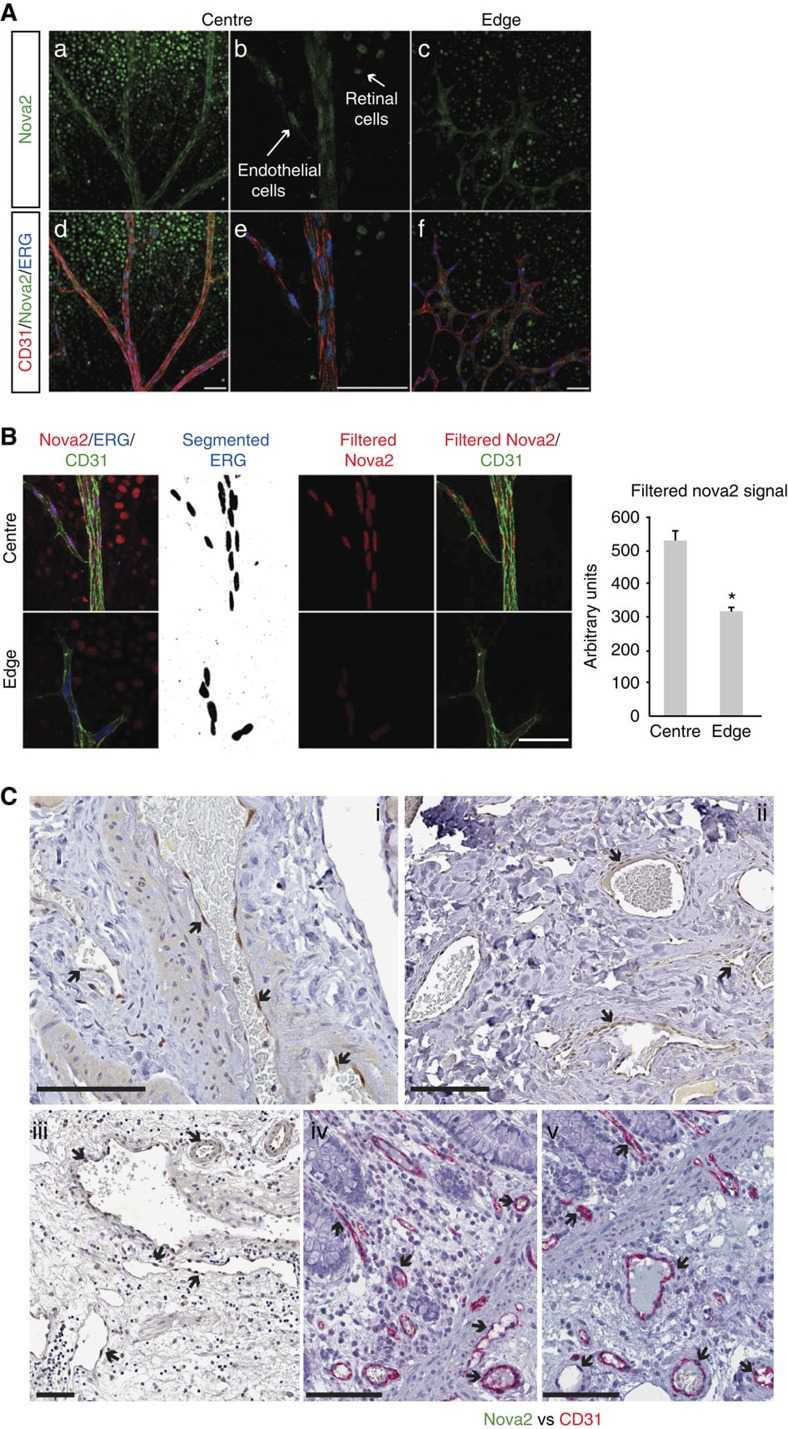
Nova2 is expressed in the vascular endothelium *in vivo*. (**a**) Immunofluorescence analysis of Nova2 (green), the endothelial markers CD31 (red) and ERG (blue, transcription factor) in whole-mounted postnatal (P6) mouse retina. Optical sections captured using confocal microscopy display large vessels in the central retina region (a,b,d,e) and sprouting ECs in the leading edge of the growing vasculature (c,f). Arrows indicate neural cells of the retina and ECs of vessels expressing Nova2 (scale bar, 50 μm). (**b**) Quantification of Nova2 signal. ERG staining has been segmented with threshold 350–4,096, and segmented images have been filtered to remove speckles and outliers (radius 25). Segmentation results have been used to filter Nova2 staining to isolate EC nuclear staining (scale bar, 50 μm). Chart shows average signals (error bars indicate mean ±s.d.; asterisks *P* value<0.05, two-tailed *t*-test assuming unequal variances; *n*=2). (**c**) IHC of Nova2 in normal human thyroid (i), skin (ii) and bladder (iii) and IHC of Nova2 and the endothelial marker CD31 in normal human colon (iv, v). Arrows indicate Nova2 nuclear staining of ECs in the blood vessels (scale bar, 100 μm).

To confirm the vascular expression of Nova2 in a more physiological context, we analysed the postnatal mouse retina, which develops a stereotypical vascular pattern following a well-defined sequence of events^22^. In the retina, we found that Nova2-positive nuclei were reduced but still present in the ECs at the sprouting front as compared with the central part of the retina where the majority of ECs of the mature vessels (arteries and veins) and capillaries were Nova2-positive (Fig. 2a,b and Supplementary Fig. 2A). In addition, we found the specific nuclear expression of Nova2 in ECs present in the vessels of different tissues, such as normal human thyroid, skin, bladder, colon and prostate (Fig. 2c and Supplementary Fig. 2B).

Collectively, these data indicate that Nova2 is not exclusively expressed in cells of the nervous system, but it is also present in ECs of different types of vessels.

### Nova2 regulates the endothelial apical–basal polarity

An important functional similarity in the development of the vascular and nervous systems is the establishment of the apical–basal polarity, as this is a crucial event for the organization of the vascular lumen and for axon guidance^23^,^24^ respectively. Notably, the partitioning-defective (Par) polarity complex is a key determinant of cell polarity in both systems^23^,^24^. The specific localization and activity of the Par polarity complex involve the association of four key components: Par3, Par6, the small GTPase Cdc42 and the atypical protein kinase C (PKCζ). In addition, the small GTPases Rac1 and Rap1 are important regulators of the Par complex during organization of the vascular lumen^23^,^25^.

**Figure 3:**
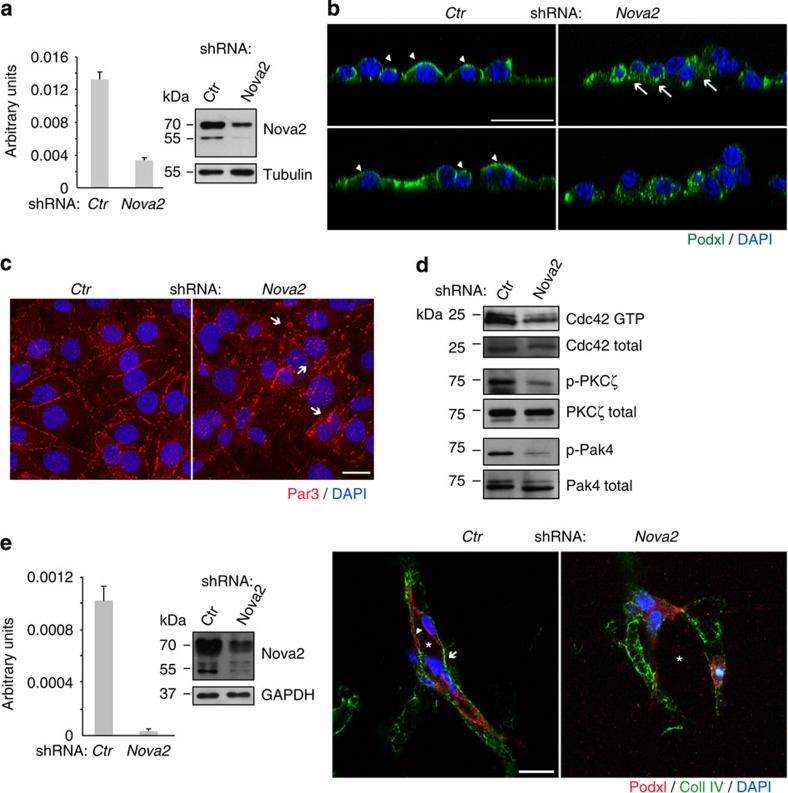
Nova2 is required for the EC polarization. (**a**) *Nova2* mRNA levels in knockdown mouse ECs (grown as confluent). The Nova2 protein level was analysed by immunoblotting using an anti-Nova2 antibody (Tubulin as loading control). (**b**) Immunofluorescence (IF) analysis of Podocalyxin (Podxl, green) and DAPI (blue) in 2D culture of control (*Ctr*) and *Nova2*-depleted ECs. Podxl is often distributed to the basal (arrows) instead of the apical surface (arrowheads) in *Nova2*-silenced ECs (confocal sections, *z* axis; scale bar, 25 μm). (**c**) IF analysis of Par3 (red) and DAPI (blue) in *Ctr* or *Nova2* knockdown ECs. Arrows indicate altered and fragmented junctional staining of Par3 (bar 20 μm). (**d**) *In vitro* pull down of GTP-bound Cdc42 in *Ctr* and *Nova2*-silenced ECs. Immunoblotting for the phosphorylation status of PKCζ and Pak4 is also shown. (**e**) Left: *Nova2* mRNA levels in knockdown HUVECs. The Nova2 protein level was analysed by immunoblotting using an anti-Nova2 antibody (GAPDH as the loading control). Right: in 3D collagen gel, control HUVECs form vascular structures with a central lumen (asterisk) and apical Podxl and basal collagen IV (Coll IV) proper localization (arrowheads and arrows, respectively), whereas *Nova2*-silenced HUVECs are not correctly polarized (scale bar, 20 μm). Error bars indicate mean ±s.d. calculated from three independent experiments (*n*=3).

To investigate the role of Nova2 in the endothelium as a possible regulator of vascular development, we generated stable *Nova2* knockdown ECs (Fig. 3a). Intriguingly, we found that depletion of *Nova2* expression impairs EC polarity. As shown in Fig. 3b, in two-dimensional (2D) cultures, *Nova2* knockdown altered the subcellular localization of the apical surface marker podocalyxin (Podxl) that was distributed all over the cell membrane and also to the basal surface. These findings prompted us to examine whether silencing of *Nova2* alters the junctional staining and/or activity of components of the Par polarity complex. *Nova2*-depleted ECs displayed impaired junctional distribution of Par3, a multiscaffold protein that promotes the assembly of the Par complex (Fig. 3c). Interestingly, *Nova2* knockdown in ECs caused reduced levels of active (GTP-bound) Cdc42 (Fig. 3d), whose association with the Par complex is induced during EC lumen formation^26^. Accordingly, we determined that phosphorylation of PKCζ, a Cdc42-GTP-activated protein^27^, is also reduced in *Nova2*-depleted ECs (Fig. 3d). Moreover, *Nova2* knockdown ECs were characterized by reduced levels of phosphorylated Pak4 (p21-activated kinase; Fig. 3d), one of the main Cdc42 downstream effectors^28^.

EC polarization correlates with lumen formation^25^,^29^. Thus, to test whether Nova2 is required for the formation of the endothelial lumen, we have carried out its knockdown in primary HUVECs that, when are cultured in three-dimensional (3D) collagen gel, rapidly organize into a network of hollow structures^30^. As shown in Fig. 3e, control HUVEC cells forming vascular-like structures were correctly polarized with Podxl and collagen IV localized to the apical and basal surfaces, respectively. On the contrary, knockdown of *Nova2* resulted in the formation of capillary-like tubular networks with irregular lumen and a non-polarized distribution of endothelial Podxl and collagen IV. These results strongly suggest that Nova2 splicing factor is crucial for EC morphogenesis.

Recently, the Par polarity complex has been shown to regulate endothelial cell-cell contacts and affect migratory behaviour^25^,^31^. In agreement with these observations we found that silencing of *Nova2* affected junctional clustering of VEC and β-catenin since the architecture of cell–cell boundaries was partially disorganized in *Nova2* knockdown ECs (Supplementary Fig. 3A). Cell polarity is also established during directional cell migration, where cytoskeletal, adhesive and signalling molecules are distributed asymmetrically. We found that *Nova2* knockdown affected the collective behaviour of migrating ECs. During wound closure, migrating ECs depleted of *Nova2* have in part lost their contacts with neighbouring cells positioned at the back (Supplementary Fig. 3B). The leading edge was more tortuous compared with control ECs (Supplementary Fig. 3B). Finally, given the junctional alterations induced by Nova2 knockdown, we tested whether endothelial permeability was also modified. We found that the permeability of confluent endothelial monolayers was indeed increased upon *Nova2* depletion (Supplementary Fig. 3C).

To comprehensively identify AS events regulated by Nova2 in the endothelium, we performed high-throughput RNAseq of two biological replicates of *Nova2* knockdown and control ECs. We used *vast-tools*^32^ to identify and quantify all major types of AS events. *vast-tools* maps RNAseq reads to comprehensive sets of annotated and novel splice junctions to derive confident estimates of the percentage of alternative sequence inclusion in a given sample. We identified 365 AS events affected by Nova2 depletion, including 188 (51.5%) cassette exons (Supplementary Fig. 4, Supplementary Table 1 and see Supplementary Methods for details). Gene Ontology (GO) analyses of AS events predicted to generate alternative protein isoforms (41% of all AS events and 64% of cassette exons; Supplementary Fig. 5) showed a significant enrichment for genes involved in cytoskeleton and cell adhesion (including tight and adherens junctions, and integrin binding), consistent with the phenotypes described above (Supplementary Table 2). In addition, the strongest enriched functional terms corresponded to chromatin remodelling and regulators, suggesting a multilayered impact of Nova2 regulation on endothelial formation. Finally, we also observed multiple GO terms related to neuronal differentiation and function (for example, neurogenesis, synapsis, axon part and calcium transport), similar to those reported for Nova-regulated genes in the brain^16^. Indeed, comparison of differentially included cassette exons in genes expressed in both neurons and ECs (see Supplementary Methods) revealed a highly significant overlap between alternative exons predicted to be regulated by Nova proteins in the brain^33^ and those showing changes in inclusion levels upon *Nova2* knockdown in the endothelium (*P*=1.93*e*^−11^, hypergeometric test; Supplementary Fig. 6; Supplementary Table 3), despite the very different approaches used in the two studies (see Supplementary Materials for details).

**Figure 4:**
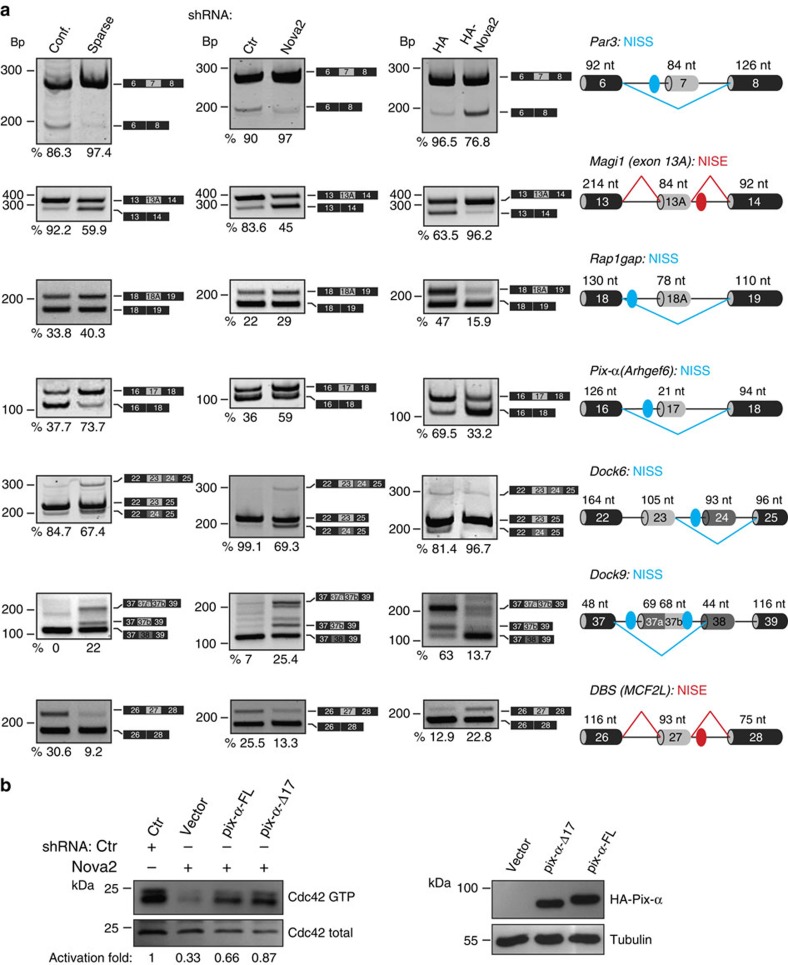
AS changes in Nova2 overexpression and knockdown ECs. (**a**) AS of the indicated Nova2 targets as determined using RT–PCR in confluent and sparse mouse ECs (left), in confluent *Nova2* knockdown ECs (middle) and in sparse ECs overexpressing HA-tagged Nova2 (right). For each gene, the schematic representations of the genomic region containing the AS exon, the transcripts generated from skipping or inclusion of the AS exon and the calculated percentage of exon inclusion are indicated. For *Dock6*, the percentage indicates the ratio between the isoform containing exon 23 (skipping exon 24) and total, whereas for *Dock9* the percentage is the ratio between the isoform containing exon 37a plus 37b and total. Grey boxes, AS exons; black boxes, constitutive exons; blue/red dots indicate YCAY clusters predicted to function as Nova silencer/enhancer. Blue/red bars indicate Nova-silenced/enhanced exon inclusion. NISS, Nova intronic splicing silencer; NISE, Nova intronic splicing enhancer. (**b**) *In vitro* pull down of GTP-bound Cdc42 in *Nova2*-silenced ECs transfected with the indicated vectors driving the expression of HA-tagged Pix-α deleted of exon 17 (Pix-α-Δ17), Pix-α containing exon 17 (Pix-α-FL) or transfected with the empty vector (vector). The Cdc42 activation fold, calculated as the ratio between GTP-bound Cdc42 and total, is shown (*Ctr* sample used as a reference value). Pix-α isoform expression was analysed by using anti-HA and Tubulin antibodies.

These newly identified Nova2 targets expanded the list of previously known targets involved in apical–basal polarity, actin polymerization dynamics and cytoskeletal remodelling, important processes associated with cell polarity, cell shape, motility and adhesion (Supplementary Tables 4 and 5). Thus, to confirm a possible molecular link between these AS events and the phenotypes described above, we analysed AS changes in selected targets using RNA extracted from *Nova2* knockdown ECs (Fig. 4a). Reduced Nova2 expression in ECs resulted in altered AS of transcripts encoding *Par3*, and regulators of Par activity or localization, including *Magi1*, which recruits Rap1 at junctions^34^, *Rap1GAP* (Rap1 inhibitor)^35^, *Pix-α*, *Dock6, Dock9* and *DBS* (Cdc42 activators)^36^,^37^,^38^ (Fig. 4a and Supplementary Fig. 7). Importantly, these AS changes parallel to those observed in the brain of *Nova2*-null mice^16^,^33^, or in two stages of brain development (Supplementary Fig. 8A) characterized by different Nova2 expression levels (Fig. 1a). We confirmed the Nova2-dependent AS of target transcripts in RNA samples extracted from *Nova2*-overexpressing ECs (Fig. 4a, Supplementary Fig. 7 and Supplementary Fig. 8B). Notably, the direction of the observed AS changes is consistent with the position of Nova2-binding sites (YCAY; Fig. 4a and Supplementary Fig. 7), as previously reported^16^.

Our data suggest that in cultured ECs Nova2 establishes EC polarity by controlling Par3 localization and by regulating the activity of Cdc42. To begin to address the functional relevance of the AS events regulated by Nova2 in ECs, we focused on the Cdc42 activator Pix-*α* (ref. 36). Our results indicate that Nova2 promotes the production of a specific Pix-*α* AS isoform lacking exon 17 (Pix-*α*-Δ17; Fig. 4a). Intriguingly, we found that Pix-*α*-Δ17 is more efficient than the Pix-*α* isoform containing exon 17 (Pix-*α*-FL) in rescuing the defect of Cdc42 activity caused by *Nova2* knockdown (Fig. 4b).

Collectively, these results provide evidence that Nova2 is required for EC polarity and that it acts by inducing AS of a set of key effectors of cell polarity.

### Nova2 promotes vascular lumen formation *in vivo*

**Figure 5:**
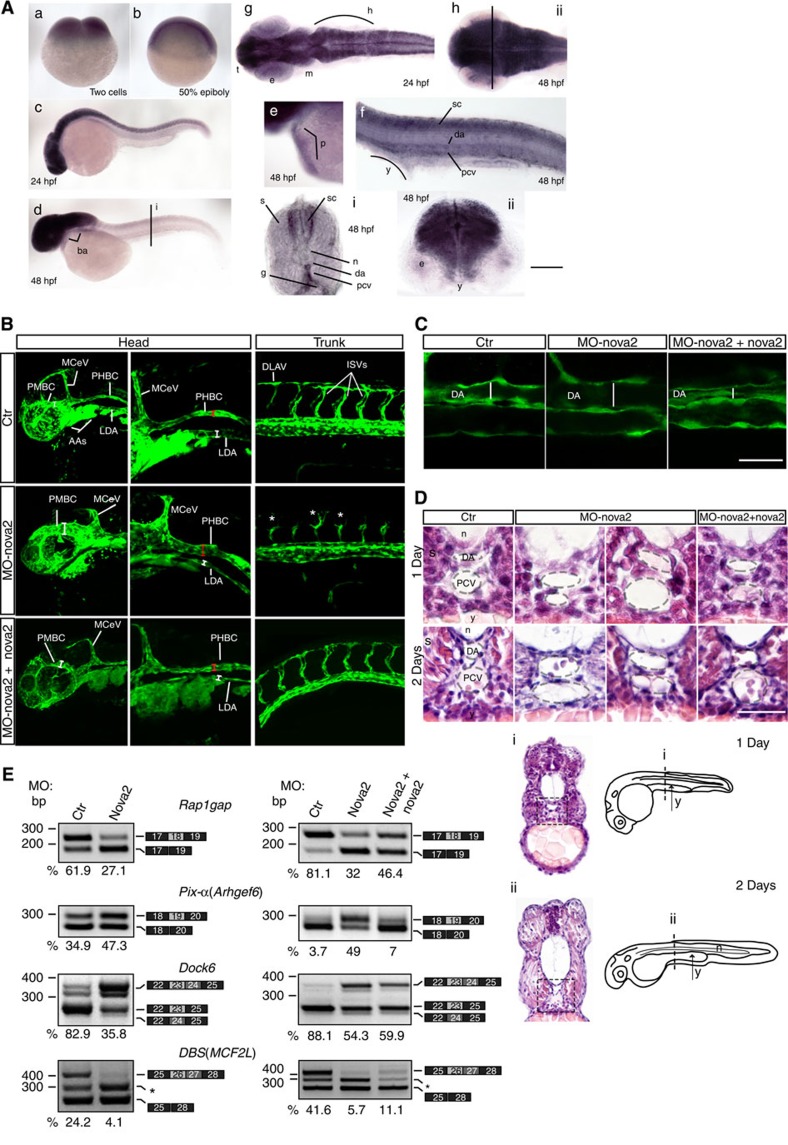
Nova2 is required for vascular lumen formation in zebrafish. (**a**) *In situ* hybridization of different zebrafish developmental stages showed *Nova2* expression in the pericardium (p in e), in the trunk vessels (f,i) of 48 hpf embryos and in the CNS of 24 (g) and 48 hpf embryos (h,ii; scale bars, 250 μm in a,b; 125 μm in c,d,g,h); 50 μm in e,f; 25 μm for vibratome sections i,ii). (**b**) Lateral views of the head and trunk of 28 hpf *Tg(fli1a:EGFP)y1* embryos, expressing the EGFP under the endothelial-specific promoter *fli1a*, injected with control (*ctr*) or morpholino against *nova2* (MO-nova2). *Nova2* knockdown results in lumen defects of lateral dorsal aorta (LDA), middle cerebral vein (MCeV) and primordial hindbrain channels (PHBC; compare red and/or white bars length between *ctr* and *nova2* morphants). The ISVs display extra-branching formation (*) and a delay in the connection with the dorsal longitudinal anastomotic vessel (DLAV). Co-injection of a morpholino-resistant zebrafish *nova2* RNA (MO-nova2+nova2) rescues the vascular defects. (**c**) Confocal analysis of the blood vessels in the trunk region of 28-hpf embryos (lateral views); *nova2* morphants display enlarged lumen (white bars) of the dorsal aorta (scale bar, 50 μm). (**d**) High magnifications of paraffin 10 μm transversal sections, stained with haematoxylin–eosin, of the trunk region of 1- and 2-day embryos (dashed black squares in i,ii) highlighted alterations of the lumen size of the dorsal aorta and PCV in more than 90% of *nova2* morphants. Abnormal phenotype was rescued by co-injection of *nova2* mRNA (scale bar, 50 μm). e, eye; g, gut; h, hindbrain; m, midbrain; n, notochord; s, somite; sc, spinal cord; t, telencephalon; y, yolk; AAs, mandibular arches; PMBC, primordial midbrain channel. (**e**) *Nova2* knockdown alters the AS of its targets (24 hpf) that is corrected by the co-injection of *nova2* mRNA (28 hpf). The percentage of exon inclusion is indicated (grey, AS exon); *Dock6* as in Fig. 4, *DBS* is the ratio 26+27/total. Asterisk, novel *DBS* AS variant containing 75 nt (exon 27b: ACGCAGGTCCTCACATCACTCTCACCCGAGTGAGATGGCTGAGCACTTCTAGTCTGTTGCAGACTAAACGCAGAG ) of intron 27.

Since EC polarity regulates vascular lumen formation, we tested whether vascular development was affected in the absence of Nova2. To this end, we investigated the role of Nova2 in the embryos and larvae of zebrafish, which constitutes a unique and powerful model to study vertebrate vascular development^39^. Importantly, the Nova2 RNA-binding domain is 94% identical between zebrafish and human^40^, and we found that a zebrafish *Nova2* orthologous gene (*nova2*) is expressed in the vasculature during development in addition to CNS (Fig. 5a). To assess the role of *nova2* in zebrafish, we performed a morpholino-mediated knockdown of its expression. To specifically visualize the developing blood vessels, we injected morpholino oligos into transgenic embryos expressing the enhanced green fluorescent protein (*EGFP*) gene under the control of the endothelial-specific promoter *fli1a* (*Tg(fli1a:EGFP)y1*)^41^. We used a morpholino targeting the start codon of zebrafish *nova2* (MO-nova2) to block translation of both maternal and zygotic *nova2* mRNAs. While embryos injected with a control morpholino displayed a normal morphology, more than 90% of *nova2* morphant embryos showed defects at the level of the forming blood vessels. Moreover, during early embryogenesis the pattern of some intersomitic vessels (ISVs) displayed extra-branching formation and a delay in the connection with the dorsal longitudinal anastomotic vessel (Fig. 5b). Confocal microscopy analysis confirmed that *nova2* knockdown resulted in altered lumen size of both cephalic vessels and of main trunk blood vessels (Fig. 5b,c). To visualize and further characterize the phenotype at the level of the dorsal aorta and posterior cardinal vein, we performed a morphological analysis using transversal paraffin sections stained with haematoxylin–eosin of embryos at different developmental stages (Fig. 5d). The analysis showed that in most of *nova2* morphants the lumen of the dorsal aorta had a larger diameter if compared with controls, through all developmental stages analysed. The lumen of the posterior cardinal vein appeared irregular along the length of the trunk, with areas of enlargement but also few restrictions (Fig. 5d). Furthermore, starting from 2 days of development, even ISVs display an enlarged lumen (Supplementary Fig. 9A). To better characterize the phenotype of *nova2* morphants at the level of ISVs during the angiogenic process, we performed an *in vivo* time lapse imaging assay. As shown in Supplementary Movies 1–3 in *nova2* morphant embryos the apical cells of some ISVs develop many more filopodia and could barely reach the dorsal longitudinal anastomotic vessel. Importantly, co-injection of *nova2* mutants with a morpholino-resistant zebrafish *nova2* mRNA rescued, in more than 60% of the injected embryos, the morphological phenotype of the vessels, confirming the specificity of the effects (Fig. 5b–d and Supplementary Movies 1–3). The abnormal phenotype of blood vessels observed in *nova2* morphants was not due to haemodynamic problems, since at 48  h post fertilization (hpf) the heart appeared normal for size, shape and beat (Supplementary Movies 4 and 5). In addition, EC proliferation and apoptosis were not significantly modified in the morphants (Supplementary Fig. 9B,C). Collectively, these data indicate that *in vivo* nova2 is required for proper vascular morphogenesis and for the formation of a correct vascular lumen.

Since Par complex members and regulators identified as Nova2 targets have putative orthologues in zebrafish, we analysed their AS using RNA extracted from *ctr* and *nova2* morphants. Of the investigated pre-mRNAs (Fig. 4a), four (*Rap1GAP*, *Pix-α*, *DBS* and *Dock6*) are alternatively spliced in zebrafish (Fig. 5e). Interestingly, in all cases nova2 knockdown alters exon inclusion levels (Fig. 5e) in the direction predicted by the position of putative Nova-binding motifs^21^ (Supplementary Fig. 10). Importantly, aberrant AS events of *nova2* morphants were rescued by co-injection of *nova2* mRNA (Fig. 5e). Of note, we found that some nova2 targets (see, for instance, *Rap1gap* and *Pix-α*, Supplementary Fig. 9C) are not expressed at the same level in the different types of vessels suggesting that the effects of the knockdown of *nova2* may have different morphological and functional consequences along the vascular tree.

**Figure 6:**
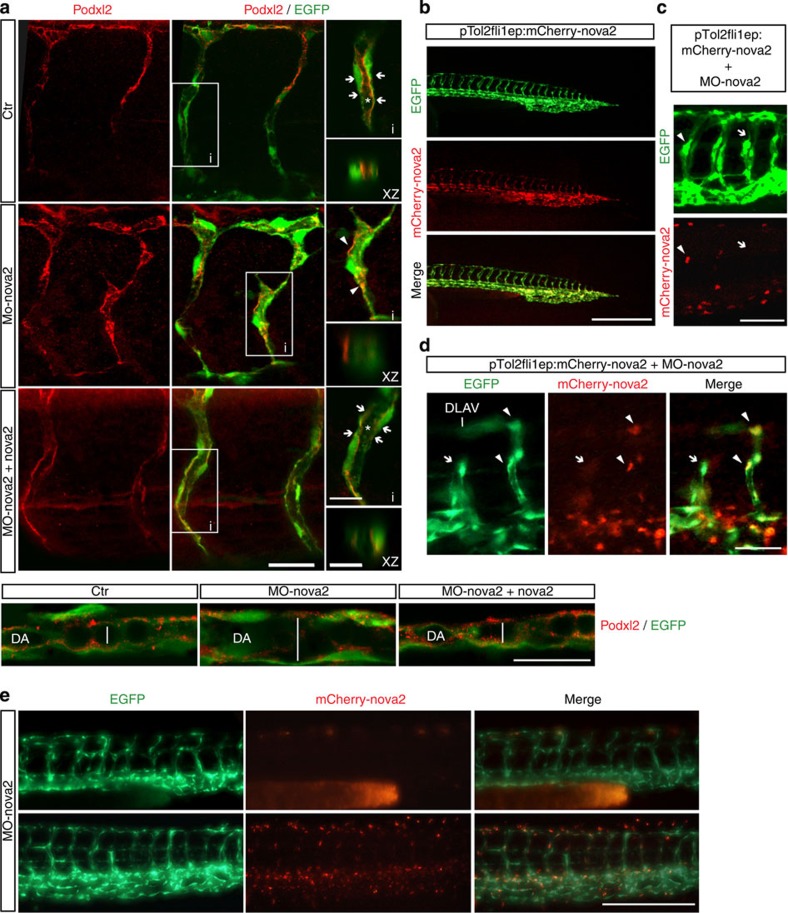
Endothelial Nova2 is essential for EC polarization. (**a**) *Tg(fli1a:EGFP)y1* zebrafish embryos were treated with control (*ctr*) or *nova2* morpholino (MO-nova2) oligos and analysed with anti-Podocalyxin antibody (Podxl2, red) in the ISVs (upper panel). Co-injection of a morpholino-resistant zebrafish *nova2* mRNA was able to rescue the apical-membrane staining (arrow) of Podxl2 in *nova2* morphant (MO-nova2+nova2; scale bar, 20 μm). Areas (i) are magnified on the right (scale bar, 10 μm). This is also visualized in the optical transverse sections (XZ, scale bar, 5 μm). Asterisk, lumen; arrowheads indicate Podxl2 mislocalization. Lower panel: Podxl2 localization in the dorsal aorta (scale bar, 50 μm). (**b**) Lateral view of the *Tg(fli1a:EGFP)y1* embryo with EGFP under the control of the endothelial-specific promoter *fli1a*. The same transgenic zebrafish embryos were co-injected at one-cell stage with a vector (pTol2fli1ep:mCherry-nova2) driving the expression of a morpholino-resistant zebrafish *nova2* mRNA fused to mCherry fluorescent protein under the *fli1a* promoter. In this case individual ECs were mosaically labelled with mCherry-nova2 expression (nova2, red) in the nucleus of some cells of the trunk blood vessels (scale bar, 250 μm). (**c**) Endothelial-autonomous rescue of *nova2* morphants. PTol2fli1ep:mCherry-nova2 plasmid was co-injected with the *nova2* morpholino oligo (MO-nova2) in one-cell stage *Tg(fli1a:EGFP)y1* embryos (bar 40 μm; arrowheads and arrows indicate vessels positive and negative for nova2, respectively). (**d**) ISV of the trunk expressing nova2 (arrowheads) showed a normal pattern and developed a correct lumen, whereas an adjacent ISV (arrows), negative for nova2 expression, was not formed properly, so that it failed to reach DLAV (scale bar, 30 μm). (**e**) Lateral view of 28-hpf transgenic embryos injected with the MO-nova2. Embryo showed in the upper row expresses in the vessel endothelium only GFP, whereas the embryo showed in the lower row expresses also a morpholino-resistant *nova2* cDNA fused with mCherry. The presence of *nova2* in the vessel endothelium is sufficient to preserve vessel morphology of *nova2* morphant embryos (scale bar, 50 μm).

To assess the apical–basal cell polarity in *nova2* morphant embryos, we analysed the localization of Podocalyxin (Podxl2). As shown in Fig. 6a, in *ctr* embryos Podxl2 is localized at the apical region of the ECs forming ISVs, while in *nova2* morphants Podxl2 is mislocalized in ECs of the ISVs. Moreover, this defect persisted at late developmental stage (Supplementary Fig. 9A). Likewise, altered Podxl2 localization was also observed in dorsal aorta of *nova2* morphants (Fig. 6a), suggesting an altered establishment of apical–basal polarity. Again, co-injection of *nova2* mRNA rescued Podxl2 localization in ECs (Fig. 6a).

Since Nova2 is expressed in neurons as well as in ECs, and these cells influence their reciprocal differentiation and development^42^, it was important to address whether the vascular phenotype of *nova2* morphants was due to alterations in the nervous or vascular systems. To do this, we used a morpholino-resistant zebrafish *nova2* cDNA, fused with the red fluorescent protein mCherry, under the control of the vasculature-specific *fli1a* promoter (Fig. 6b). The vascular defects in *nova2* morphants were restored by the mosaic transient expression in the vascular endothelium of morpholino-resistant *nova2* cDNA (Fig. 6b–d). Moreover, by generating a zebrafish transgenic line, which stably expresses morpholino-resistant *nova2-mCherry* in the vascular endothelium, we found that injection of MO-nova2 did not significantly alter blood vasculature morphology (Fig. 6e), indicating that the vascular phenotype of *nova2* morphants is cell autonomous.

To independently validate our findings, we used CRISPRs genome engineering^43^ to generate genetic *nova2* mutant fish (Supplementary Fig. 11A and see Methods for details). The characterization of *nova2* mutants strongly supported our results and conclusions obtained using MO-mediated *nova2* knockdown (Supplementary Fig. 11). In particular, *nova2* homozygous mutants displayed overlapping defects with *nova2* morphants, such as shortening of the anteroposterior body axis, reduced head size, curved trunk and slight pericardial oedema (Supplementary Fig. 11B). Moreover, we observed that *nova2* mutants do not develop the swim bladder and display a strong haemorrhagic phenotype visible both at the level of the head and trunk (Supplementary Fig. 11C,D). Notably, similar to Nova knockout mice^44^,^45^, zebrafish *nova2* mutant embryos displayed paralysis. In particular, they have severe difficulty in swimming (probably because of motor–neuronal dysfunction) and died 7–10 days post fertilization, whereas there was no abnormal phenotype in *nova2* heterozygote mutant embryos. Confocal microscopic analyses performed on the *nova2* mutants transgenic for *kdr:EGFP* showed that many blood vessels had an enlarged lumen size, both in the head and in the trunk (Supplementary Fig. 11E), as observed in *nova2* morphant embryos. Similar findings were obtained by histological analysis (Supplementary Fig. 11F). Finally, *nova2* mutants displayed AS changes (Supplementary Fig. 11G) that are comparable to those in *nova2* morphants.

Collectively, our results show that Nova2 controls the development of the vascular system *in vivo* by modulating endothelial polarity and lumen formation.

## Discussion

Here we report that AS regulation orchestrates some important aspects of EC biology. In particular, our data demonstrate that the AS factor Nova2, known to be neural cell-specific^16^, is also expressed in the vascular endothelium and plays a relevant role in vascular morphogenesis.

In spite of its importance, our current understanding of the mechanisms underlying vascular tubulogenesis is only beginning to be unravelled^23^,^25^,^29^. We have shown that Nova2 acts as a post-transcriptional regulator of the molecular mechanisms involved in the organization of the vascular lumen. In zebrafish, depletion and genetic knockout of *nova2* prevents proper formation of the lumen of blood vessels and also results in defects in EC polarization. Interestingly, in avascular organisms, such as *Drosophila melanogaster*, the Nova homologue (Pasilla, *ps*), is not expressed in the brain, but instead is expressed at high levels in salivary glands and several other non-neuronal tissues^46^,^47^. Notably, *ps* mutants have altered apical secretion and are characterized by developmental defects of the salivary gland including regions of lumen alteration^46^. This type of morphology, with a severely altered lumen, is somehow reminiscent of the morphology of the zebrafish vasculature observed on *nova2* depletion. Recently, several regulators of salivary gland lumen formation (such as Cdc42, Pak proteins and cadherins) have been identified in *Drosophila*^48^. Intriguingly, they are also implicated in vascular lumenogenesis^23^ and, more importantly, regulators of their activity and localization are known Nova2 AS targets^33^.

Our data show that, in cultured ECs, Nova2 establishes EC polarity by controlling Par3 localization, the activity of Cdc42 and the phosphorylated state of PKCζ. Notably, signalling downstream of Cdc42 is impaired in the absence of Nova2, as the active form of PKCζ and Pak4 are reduced in Nova2-depleted ECs. Interestingly, Pak4 phosphorylation correlates with EC lumen formation and RNA interference-mediated suppression of *Pak4* strongly inhibits these processes^26^. Similarly, depletion of *Nova2* impairs the establishment of EC polarity and the organization of the vascular lumen. These defects are associated with aberrant AS of pre-mRNAs encoding factors belonging to the Par polarity complex and its regulators.

Par complex and downstream effectors play important roles in regulating cell–cell adhesions^25^, in controlling the organization of the microtubule cytoskeleton^49^, and in promoting directional, collective cell migration^31^. Strikingly, we found that downregulation of *Nova2* affected the architecture of cell–cell boundaries and the behaviour of migrating ECs. In particular, in wound closure *Nova2*-depleted ECs failed to move in a cohesive manner with the lack of coordination in the direction of cell movement suggesting that, in addition to its role on lumen formation, Nova2 expression might also control adhesion signals and the directional migration of ECs.

Our current lack of knowledge of the functional implications of most AS events makes it difficult to interpret the global impact of the AS changes we have identified. Moreover, it is plausible that additional AS events are regulated by Nova2 in ECs. Nevertheless, we found that the alteration in Cdc42 activity in *Nova2* knockdown ECs is preferentially reverted by overexpression of a specific AS isoform of Pix-*α* (Pix-*α*-Δ17) regulated by Nova2, indicating that AS of this gene plays an important role in Cdc42 activation.

We found Nova2-dependent AS regulation of zebrafish orthologous genes encoding polarity regulators. Among these, there are well-characterized activators (*Pix-α, Dock6* and *DBS*) of Cdc42, which—in turn—plays an essential role in controlling lumen formation *in vitro* and *in vivo*^26^,^48^,^50^,^51^,^52^.

Remarkably, Nova2-regulated pre-mRNA targets encode proteins that interact with each other^16^, suggesting that Nova2 regulates a network of apical–basal polarity regulators and that AS plays an important role in affecting physical interactions between these factors during the organization of the vascular lumen. Hence, the phenotypic changes that we observed on *Nova2* knockdown are likely the integrated effects of several AS changes that may act in a coordinated and non-redundant manner.

On the basis of the fact that Nova2 affects both neural and vascular cell processes, we suggest that Nova2 is a novel member of the ‘angioneurins' family^3^,^4^. Interestingly, Nova2 is the only member of the angioneurin family defined so far that functions as post-transcriptional regulator. Malfunctioning or imbalance in angioneurin signalling contributes to various neurological disorders, indicating that non-neuronal defects contribute to these diseases^3^. In line with this, in Alzheimer's disease patients' vascular dysfunction can be observed before the onset of the disease, suggesting that vascular alterations might causally contribute to disease initiation or progression^53^. Notably, significant AS changes associated with decreased Nova activity were reported in Alzheimer's patients^54^. Since Nova2 is an autoimmune target in a severe neurodegenerative disorder paraneoplastic opsoclonus myoclonus ataxia (POMA)^55^, an obvious question is whether POMA patients, in addition to displaying neurological symptoms, also develop vascular abnormalities.

## Methods

### Cell culture

ECs were isolated by dissection and dissociation with collagenase type I (Roche, 1.5 mg ml^−1^), DNase (Roche, 25 μg ml^−1^) at 37 °C for 1 h and by passing through a 40-μm cell strainer. ECs were immortalized by infecting them with a retrovirus expressing the polyoma middle-sized T antigen. VEC-null and VEC-positive were grown as sparse or confluent by placing 500,000 cells in 100- and 35-mm Petri dishes, respectively.

Culture medium of mouse ECs, VEC-null and VEC-positive ECs was DMEM (GIBCO) with 20% fetal bovine serum (FBS; HyClone), glutamine (2 mM, Sigma-Aldrich), penicillin/streptomycin (100 U l^−1^, Sigma-Aldrich), sodium pyruvate (1 mM, Sigma-Aldrich), heparin (100 μg ml^−1^, from porcine intestinal mucosa; Sigma-Aldrich) and EC growth supplement (5 μg ml^−1^, made from calf brain; complete culture medium). HUVECs were isolated from umbilical vein by treatment with Collagenase (Roche, 0.1%, for 30 min at 37 °C) and were cultured in MCDB 131 with EC supplements.

### Mouse ES cell culture

To obtain endothelial differentiation of mouse ES cells, cells were mildly trypsinized and suspended in Iscove's modified Dulbecco medium with 15% FBS, 100 U ml^−1^ penicillin, 100 μg ml^−1^ streptomycin, 450 μM monothioglycerol, 10 μg ml^−1^ insulin, 50 ng ml^−1^ human recombinant VEGF-A165 (Peprotech Inc.), 2 U ml^−1^ human recombinant erythropoietin (Cilag AG) and 100 ng ml^−1^ human basic fibroblast growth factor (bFGF) (Genzyme). Cells were seeded in Petri dishes and cultured for 4 or 7 days at 37 °C with 5% CO_2_ and 95% relative humidity.

### Lentivirus production and transduction

GIPZ Lentiviral Nova2 short-hairpin RNAs (shRNAs) were obtained from Open Biosystems, while pLenti-GIII-CMV-humanNova2-HA from THP Medical Products. HEK293T (American Type Culture Collection, CRL-1573) cells were seeded in DMEM-HIGH supplemented with 10% FBS without antibiotics in 60-mm Petri dishes (one Petri per infection). The day after, cells at 60–70% confluence were transfected (calcium phosphate transfection method) using these quantities of DNA: 5 μg of packaging plasmid, 5 μg of envelope plasmid and 20 μg of Nova2 vectors. After 18 h, the medium was replaced with growth medium DMEM supplemented with 20% FBS and 1% penicillin/streptomycin. Cells were incubated for 24 h and the medium containing the lentiviral particles was harvested, filtered using a 0.45-μm filter unit and used to infect the cells.

For viral transduction, mouse EC cells were seeded in 100-mm Petri dishes and were infected at 70% of confluence. Cells were incubated overnight with the viral supernatant supplemented with 0.2 mM proline and polybrene (final concentration 8 μg ml^−1^; Sigma). After 48 h puromycin selection (3 μg ml^−1^) was started and it was continued until all non-infected control cells died (typically, 5 days).

### 2D culture

Mouse ECs transduced with lentiviral vectors carrying shRNA for Nova2 were seeded in 35-mm Petri dishes coated with Gelatin (Difco) 0.1% and cultured for 72 h. The splitting ratio was such that confluence was reached overnight after seeding. For immunofluorescence, ECs were fixed with 4% paraformaldehyde (PFA) and then permeabilized with 0.5% Triton X-100 for 10 min. Blocking (1 h), primary (overnight) and secondary (1 h) antibodies were diluted in PBS with 2% BSA. The following primary antibodies were used: anti-Par3 (1:100 Millipore), anti-Podocalyxin (1:100 R&D), anti-VE-cadherin (1:100 C-19, sc-6458, goat, Santa Cruz Biotechnology) and anti-β-catenin (1:50 BD Transduction Laboratories). Secondary antibodies for immunofluorescence were donkey antibodies to the appropriate species conjugated with Alexa Fluor 488, 555 or 647 (dilution 1:200 or 1:400).

For imaging, charge-coupled device camera on epifluorescence microscope (Leica) or Leica TCS SP2 confocal microscopy were used. ImageJ (NIH) was employed for data analysis. Figures were assembled using Adobe Photoshop and Adobe Illustrator. Only adjustments of brightness and contrast were used in the preparation of the figures. For comparison purposes, different sample images of the same antigen were acquired under constant acquisition settings.

### Immunoblot analysis

Cells were lysed in Laemmli buffer and proteins were separated using SDS–PAGE and analysed with western blotting. The following primary antibodies were used: anti-Nova2 (1:200 Santa Cruz Biotechnology, C-16), anti-GAPDH (1:5,000 Abcam; 1:50,000 AbFrontier), anti-Tubulin (1:100,000 Sigma-Aldrich), anti-total-Pak4 (1:1,000 Cell Signaling), anti-phospho-Pak4 (1:1,000 Cell Signaling), anti-haemagglutinin (HA; 1:1,000 Covance), anti-total-PKCζ (1:1,000 Abcam), anti-phospho-PKCζ (1:1,000 Cell Signaling) and anti-Actin (1:500 Santa Cruz Biotechnology). The following secondary antibodies linked to horseradish peroxidase (Jackson ImmunoResearch) were used: anti-Mouse (1:10,000), anti-Goat (1:5,000) and anti-Rabbit (1:10,000). Immunostained bands were detected using the chemiluminescent method (Pierce).

### Retinal immunohistochemistry

All animal work using mice was conducted in accordance with the Swedish Animal Welfare Board at the Karolinska Institutet, Stockholm, Sweden. Eyes retrieved from pups at P6 were fixed in cold 100% MeOH and stored at −20 °C before dissection. After dissection, retinas were incubated in 5% donkey sera, 1% BSA and 0.5% Triton X-100 in PBS overnight and then incubated with antibodies towards Nova2 (Santa Cruz Biotechnology, C-16), PECAM (BD Bioscience, MEC13.3) and ERG (Abcam, ab92513) overnight. For secondary detection, retinas were incubated with fluorescently conjugated antibodies (Jackson ImmunoResearch) and mounted flat with ProLong Gold (Invitrogen).

### Immunohistochemistry

All procedures involving human samples were approved by the Istituto Europeo di Oncologia (IEO) Ethical Committee. When possible, a written informed consent for research use of biological samples was obtained from all patients, and the research project was approved by the Institutional IEO Ethical Committee. Immunohistochemistry (IHC) was performed using 3-μm sections from formalin-fixed and paraffin-embedded tissue samples. Samples were rehydrated through xylene and graded alcohols. Antigen retrieval was accomplished using 10 mM sodium citrate, 0.05% Tween20, pH 6.0. Samples were then incubated with 3% H_2_O_2_ for 5 min, followed by 30 min of blocking in 2% BSA and 0.05% Tween20, and then by the incubation with goat anti-Nova2 (1:100 Santa Cruz Biotechnology, C-16) overnight at 4 °C in 2% BSA and 0.02% Tween20. Immunocomplexes were visualized with LSAB+System-HRP, DAKO (K0690) and acquired with the Aperio ScanScope system. Slides were counterstained with haematoxylin for histological evaluation. Double IHC was performed as follows: antigen retrieval was accomplished using 1 mM EDTA, 0.05% Tween. Samples were then incubated with 3% H_2_O_2_ for 5 min, followed by 30 min of blocking in 2% BSA and 0.02% Tween20, and then were incubated with a mix of goat anti-Nova2 (1:200) and mouse anti-CD31 (1:60 DAKO, Clone JC70A) in 2% BSA and 0.02% Tween20 for 2 h at room temperature. Immunocomplexes were visualized with an anti-goat horseradish peroxidase (HRP) detection system (Goat-on-Rodent HRP-Polymer, Biocare Medical), for 30 min at RT, followed by incubation with a Goat Anti-Mouse AP Polymer detection system (MACH 2 Mouse AP-Polymer Biocare Medical) for 30 min at RT. CD31 was visualized in red using the Vulcan Fast Red Chromogen (Biocare Medical) and Nova2 was visualized in green using the Vina Green Chromogen Kit (Biocare Medical) according to the manufacturer's protocol.

### Migration assay

To analyse cell migration, the wound-healing technique was used. Briefly, confluent EC monolayers on a tissue culture dish were wounded by manually scratching with a pipette tip after an overnight starving, washed with starving medium and incubated at 37 °C for 8 h in complete media containing Mitomycin C (4 μg ml^−1^). The wound-induced cell migration was followed by staining with fluorescent phalloidin (10 μM, Sigma).

### Paracellular flux

Mouse ECs were seeded on 0.4-μm pore size Transwell Permeable Supports (Corning) cultured in complete culture medium before assaying permeability. Next, fluorescein isothiocyanate dextran (70 kDa; Sigma) was added to the medium of the transwell apical compartment. At different times of incubation, a 50-μl aliquot of the medium was collected from the basal compartment, and the paracellular tracer flux was measured as the amount of fluorescein isothiocyanate dextran in the medium using a fluorometer.

### 3D culture in collagen gels

HUVECs were transduced with GIPZ lentiviral vectors (Open Biosystems) carrying shRNA for Nova2 or control shRNA. Control and *Nova2* knockdown HUVECs were cultured in 3D collagen gel. The final cell density in collagen (3.5 mg ml^−1^ final concentration collagen type1 from rat tail, High Concentration, BD Biosciences) was 5 × 10^5^ cells ml^−1^. Culture medium was 199 with 1% FCS, Insulin-Transferrin-Selenium supplement (Life Technologies), 50 ng ml^−1^ phorbol myristate acetate, 50 μg ml^−1^ ascorbic acid, 40 ng ml^−1^ VEGF and 40 ng ml^−1^ bFGF. For confocal microscopy, 190 μl cell suspension in collagen was used for each microwell (μ-slide 80826, Ibidi, Germany). 3D cultures were fixed with 3% PAF for 35min, quenched with 75mM NH_4_Cl and 20mM glycine in PBS, pH 8, for 10min and blocked with 0.7% FSG and 0.3% Triton X-100 PBS (blocking buffer) for 30min. Primary and secondary antibodies were incubated overnight at 4°C. Primary antibody contained 5% donkey serum. Washes in blocking buffer were performed over the course of a day at room temperature. For immunofluorescence, the following primary antibodies were used: anti-Podocalyxin (R&D, 1:400) and anti-Coll IV (AbD Serotech, 1:200). Secondary antibodies for immunofluorescence were donkey antibodies to the appropriate species conjugated with Alexa Fluor 488, 555 or 647 (dilution 1:200 or 1:400).

### Plasmids

Mouse Pix-*α*-FL fused to HA-tag was amplified with primers SG57-F and SG56-R (Supplementary Table 9) and was cloned in pcDNA3.1(+) vector (Invitrogen), whereas Pix-*α*-Δ17 was generated by PCR-mediated mutagenesis of Pix-*α*-FL (using primers SG55-F/-R and SG56-F/-R). All constructs were verified by sequencing.

### RNA extraction and RT–PCR

Total RNA was isolated using the RNeasy Mini Kit (QIAGEN) according to the manufacturer's instructions. After treatment with DNase (Ambion), 2–3 μg of RNA was retro-transcribed with mix of d(T)_18_ oligos and random hexamers or gene-specific primers (Supplementary Table 10) and Superscript III RT (Invitrogen). An aliquot (1/20th) of RT was then PCR-amplified. For qPCR, an aliquot of the RT reaction was analysed with QuantiTect SYBR Green PCR (QIAGEN) by using LyghtCycler 480 (Roche). Target transcript levels were normalized to those of reference gene. The expression of each gene was measured in at least three independent experiments. All primers are listed in Supplementary Tables 6 and 7. AS bands were quantified using densitometric analysis.

### Pull down of GTP-bound Cdc42

Nova2-depleted and control mouse ECs cultured in 100-mm Petri dishes were analysed for Cdc42 activity by using the Cdc42 Activation Assay Kit (Abcam) according to the manufacturer's instructions. For experiments with *Pix-α* minigenes, cells were transfected with Lipofectamine 3000 (Invitrogen) and grown to reach confluence before the beginning of the pull down.

### Zebrafish strains and maintenance

Zebrafish (*Danio rerio*) from wild-type *AB* and transgenic *Tg(fli1a:EGFP)y1* (ref. 41) strains were maintained and bred according to the national guidelines (Italian decree ‘4 march 2014, n.26'). All experimental procedures were approved by the FIRC Institute of Molecular Oncology Institutional Animal Care and Use Committee.

### *In situ* hybridization on zebrafish embryos and sections

Zebrafish *nova2* cDNA was amplified using PCR (primers CG13F and CG13R in Supplementary Table 9) and cloned into pCRII-TOPO vector (Invitrogen). The antisense RNA probe was generated using T7 RNA Polymerase and digoxigenin-labelled UTPs (kit from Roche) and was purified with the RNeasy Mini Kit (QIAGEN) according to the manufacturer's instructions. Whole embryos of different developmental stages were fixed overnight in 4% PFA, washed in PBS and pre-incubated for 2 h at 63 °C in hybridization buffer. Next, the *nova2* probe was added to the mix and incubated overnight at 63 °C. Embryos were then washed in SSC buffer and pre-incubated for 2 h in blocking medium at room temperature. A ratio of 1:2,000 anti-DIG antibody conjugated with alkaline phosphatase (Roche) was added and incubated overnight at 4 °C. After several PBS washes, embryos were incubated in NBT/BCIP staining buffer. Stained embryos were then equilibrated in glycerol 85% in PBS overnight at 4 °C and were observed with a stereomicroscope equipped with optic fibres. To prepare 50-μm transversal sections of 48- hpf embryos, previously stained by *in situ* hybridization, we cut them in PBS with a vibratome after inclusion in 5% low-melting agarose. Sections obtained were equilibrated and mounted in glycerol 85% in PBS on glass slides and observed under a Nikon Upright microscope. All images were acquired with high-resolution digital cameras (Nikon).

To detect the mRNA expression of *Rap1gap* and *Pix-α*/*Arhgef6* genes and GFP, we use 5-μm paraffin sections of the head of 48-hpf *Tg(fli1a:EGFP)y1* embryos fixed overnight with 4% PFA in PBS. To orientate embryos in the proper way before including in paraffin and cutting microtome sections, we have pre-included them in 1% low-melting agarose (in PBS) under a dissecting microscope with optic fibers, using plastic base moulds of 7 mm of diameter. Paraffin was removed with xylene and sections were rehydrated through graded EtOH washes, permeabilized with Proteinase-K and HCl 2 N and were incubated overnight at 64 °C in hybridization mix with DIG-labelled LNA probes for *Rap1gap* (5′-TTCACCTCTTCACAGACAAGCT-3′) and *Pix-α/Arhgef6* (5′-TAGACGTAGAGGTGTTGGACT-3′) (designed and produced by Exiqon). Sections were then treated overnight at 4 °C with an anti-DIG antibody (1:2,000 Roche) conjugated with AP and stained with NBT/BCIP. After several washes in PBS, embryo sections were incubated overnight at 4 °C with mouse anti-GFP antibody (Upstate, 1:200) and labelled using the *Vina Green Chromogen kit* (Biocare Medical).

### Zebrafish haematoxylin–eosin staining and immunofluorescence

Agarose was used to embed 24- and 48-h zebrafish embryos before including in paraffin and cutting microtome 10-μm sections. Sections were stained with haematoxylin– eosin to assess the histological features. For immunofluorescence, paraffin was removed with xylene and the sections were rehydrated in graded alcohol. Antigen retrieval was carried out using preheated target retrieval solution (pH 6.0) for 45 min. Tissue sections were blocked with FBS serum in PBS for 90 min and incubated overnight with the following primary antibodies: GFP (1:100 Millipore), Phosphohistone H3 (PHH3, 1:100 Millipore), Caspase3 (1:100 Cell Signaling) and Podocalyxin (Podxl2 1:200, kindly provided by Heinz-Georg Belting). Alexa Fluor-conjugated antibodies were used for the immunodetection, and all sections were counterstained with 4,6-diamidino-2-phenylindole (DAPI) and visualized using a Confocal microscope (Leica SP2).

### Morpholino injections and RT–PCR of zebrafish embryo

Zebrafish embryos at one- to two-cell stage were injected with 6.7 ng of an ATG-morpholino antisense oligonucleotide (Supplementary Table 8), designed to block translation of the zebrafish *nova2* gene (ENSDARG00000017673). To analyse the kinetics and the pattern of formation of the ISVs, we mounted in 1.2% low-melting agarose in E3 water *Tg(fli1a:EGFP)y1*-dechorionated embryos at 22 hpf, previously anaesthetized using 15 mg l^−1^ of tricaine (Sigma). Image acquisition was performed overnight every 10 min with a × 40 water immersion objective on a Leica TCS SP2 confocal microscope. Confocal stacks from each time point were processed for maximum intensity projections and, subsequently, movies were generated with the Leica LCS software. To observe and evaluate the shape and function of the heart, we mounted under a stereomicroscope 48-hpf dechorionated embryos in 3% methyl-cellulose in E3 water, previously anaesthetized using 15 mg l^−1^ of tricaine (Sigma). Using high-resolution digital camera (Nikon), we acquired 13-s movies from both control and *nova2* morphant embryos. Total RNA was extracted from 24-hpf pooled embryos with TRIzol reagent (Invitrogen), purified with the RNeasy Mini Kit (QIAGEN) and retro-transcribed with d(T)_18_ oligo or gene-specific primer and Superscript III RT (Invitrogen) and then analysed in PCR for AS modification of *nova2* targets. To rescue morphological and vascular defects due to the knockdown of *nova2*, we amplified with primers CG30F/CG13R (Supplementary Table 9) by using RT generated from *Tg(fli1a:EGFP)y1* embryos, a morpholino-resistant zebrafish *nova2* cDNA, with six mismatches in the pairing region with the morpholino, which, however, do not alter the amino-acid sequence of the translated protein. We cloned this cDNA in pCRII-TOPO vector (Invitrogen) and we transcribed *in vitro* the capped mRNA using SP6 mMessage mMachine kit (Ambion). Co-injection in one- to two-cell stage embryos of 6.7 ng of MO-nova2 and 160 pg of each *nova2* mRNAs was performed. The molecular and morphological changes of *nova2* morphants were scored at 28 hpf.

### Generation of new fish line for endothelial-specific rescue

To drive Nova2 expression selectively in the zebrafish vascular endothelium, we cloned the morpholino-resistant zebrafish *nova2* cDNA into the pTolfli1epCherryDest vector^56^ in frame with mCherry at the N terminus under the endothelial *fli1a* enhancer/promoter flanked by Tol2 transposable elements (*pTolfli1:nova2-CherryDest*). Fertilized eggs from *casper* x *Tg(fli1a:EGFP)y1* mutant/transgenic line were injected with 2 nl of a mixture containing 25 ng μl^−1^ of the circular plasmid *pTolfli1:nova2-CherryDest* and 25 ng μl^−1^ of T2 transposase mRNA. Injected embryos were selected for simultaneous expression of GFP and mCherry in the vessels, raised to adulthood and crossed with *casper* × *Tg(fli1a:EGFP)y1* fish. F1 embryos were observed under a fluorescent dissecting microscope from days 1 to 5 after fertilization to select carrier fish of the new mutant/double transgenic line *casper* × *Tg(fli1a:EGFP)y1*+*Tg(fli1:nova2-mCherry*).

To assay the capability of *nova2* mRNA to rescue *nova2* morphant phenotype specifically in the vessel endothelium, we performed two experiments. Co-injection of 6.7 ng of MO-nova2, 50 pg of the rescuing DNA construct *pTolfli1:nova2-CherryDest* and 120 pg of Tol2 transposase mRNA was performed directly into the cytoplasm of one-cell stage *casper × Tg(fli1a:EGFP)y1* embryos. The phenotype of the vessels of the resulting ‘mosaic' embryos was scored at 28 hpf. Alternatively, injection of 6.7 ng of MO-nova2 was performed into one-cell stage *casper* × *Tg(fli1a:EGFP)y1*+*Tg(fli1:nova2-mCherry)* embryos. The phenotype of all the vessels of the resulting embryos was scored at 28 hpf.

### Immunofluorescence staining of zebrafish embryos

Zebrafish embryos from the *Tg(fli1a:EGFP)y1* strain at 48 hpf were dechorionated and fixed in 2% PFA in PBS, overnight at 4 °C. Embryos were then washed four times for 5 min in PBST (PBS+0.1% Tween20). Permeabilization in PSBT+0.5% Triton X-100 was performed for 30 min at room temperature. Embryos were then blocked in a solution of PBST+0.5% Triton X-100, 10% normal goat serum and 1% BSA for 2 h at room temperature. Embryos were incubated with primary antibodies in blocking solution overnight at 4 °C. Successively, embryos were washed six times in PBST over 4 h at room temperature and then incubated with secondary antibodies in blocking solution, overnight at 4 °C. Embryos were washed finally six times in PBST over 4 h at room temperature and equilibrated in glycerol 85% in PBS. The following antibodies were used: mouse anti-GFP (1:2,000 Millipore); rabbit anti-podocalyxin (1:150 kindly provided by Heinz-Georg Belting); rabbit anti-mouse Alexa-488-conjugated IgG (1:400); and goat anti-rabbit Alexa 546-conjugated IgG (1:400). For the microscope analysis, we mounted on slides the trunk and tail regions dissected from five to six embryos of each samples. Images were taken with a Leica TCS SP2 confocal microscope, using oil-immersion objective × 40.

### Generation of nova2 zebrafish mutant by CRISPR/Cas9

To identify the best target site and to design the single guide RNA (gRNA), we submitted to the ZiFiT targeter website (http://zifit.partners.org/ZiFiT) the sequence of the first exon of the zebrafish *nova2* gene. Oligonucleotides corresponding to the target region (see Supplementary Fig. 11A and Supplementary Table 11) were annealed and cloned into the pDR274 gRNA expression vector (Addgene, 42250) immediately upstream of the crRNA:trascRNA backbone. The *nova2* genomic target sequence starts with two GG nucleotides at the 5′ end for efficient transcription from the T7 promoter and ends with the protospacer-adjacent motif. The correct position of the target sequence into the pDR274 plasmid was verified through sequencing, using M13-Rev primer (Supplementary Table 11). The *nova2* gRNA was *in vitro* transcribed and purified from DraI-digested plasmid as template, using the Maxiscript T7 Kit (Life Technologies). Capped and polyadenylated *Cas9* mRNA was *in vitro* transcribed and purified from 1 μg of PmeI-digested pMLM3613 Cas9 expression vector (Addgene, 42251) as a template, using the mMessage mMachine T7 ULTRA kit (Life Technologies).

A volume of 2 nl of a solution containing ∼13 ng μl^−1^ of *nova2* gRNA and ∼300 ng μl^−1^ of *Cas9* mRNA was co-injected in one-cell stage zebrafish embryos. On the next day, only embryos with a normal morphological phenotype were allowed to grow. To evaluate the efficiency of Cas9 nuclease activity, genomic DNA was extracted from 48-hpf single injected embryos and the *nova2*-targeted genomic locus was amplified from genomic DNA of each embryo (primers n2-locus-F and n2-locus-R in Supplementary Table 11). PCR products were then processed for the T7 Endonuclease I (T7EI) assay. PCR products were denaturated at 95 °C and rapidly re-annealed using a PCR thermocycler. PCR products were then incubated for 20 min with T7EI enzyme at room temperature, and the digestion products were visualized on 2% agarose gels. We found that 95% of the injected embryos had mutations at the level of the *nova2* target site.

To analyse the kinds of mutations obtained at the level of the *nova2* locus, we sequenced PCR products, cloned into pCRII-TOPO plasmid using the TOPO TA cloning kit (Life Technologies), amplified from genomic DNA extracted from fin-fragments of 2-month fishes. Among these fishes, we isolated those carrying the mutation also in the germline and subsequently we selected from their progeny fishes with nonsense or missense mutations in the *nova2* locus. A male carrying a nonsense mutation was crossed with a female from the *Tg(kdrl:EGFP)* line to generate a double *EGFP* transgenic-*nova2* mutant line expressing the EGFP reporter in vascular ECs and allowing to characterize the vascular phenotype of *nova2* mutants.

### o-dianisidine staining

For histochemical staining of haemoglobin, 72 hpf live embryos were incubated with o-dianisidine staining solution (40% ethanol, 0.01 M sodium acetate, 0.65% H_2_O_2_, 0.6 mg ml^−1^ o-dianisidine (Sigma, D-9143) for 15 min. Embryos were then washed with PBS, post-fixed in 4% PFA in PBS overnight at 4 °C and stored in 85% glycerol for microscope analysis.

### RNAseq and splicing analysis

RNAseq was conducted on two control and two *Nova2*-depleted ECs. Samples were sequenced on Illumina HiSeq2500 (average of ∼93 million, 100-nucleotide (nt) paired-end reads for each run). We employed *vast-tools*^32^ to identify and quantify all major types of AS events, including single and multiple cassette exons and microexons, alternative 5′ and 3′ splice sites and alternatively retained introns, from each RNAseq sample. *vast-tools* map reads to comprehensive sets of exon–exon junctions (EEJs) and exon–intron junctions (EIJs) to derive alternative sequence inclusion levels (PSIs, ‘Percent Spliced In', for exons; PIR, ‘Percent Intron Retention', for introns)^57^. We then compared the two replicates of *Nova2* knockdown ECs versus control ECs. Differentially regulated AS events were defined as those showing an absolute ΔPSI⩾15 between knockdown and control means and a ΔPSI⩾5 between the ranges of the two groups. Only AS events with a minimum read coverage in all four samples were compared, which was defined as:

- For cassette exons (except for those quantified using the microexon pipeline, see Irimia *et al*. 32 for details): (i) ⩾10 reads mapping to the sum of exclusion EEJs or (ii) ⩾10 reads mapping to one of the two inclusion EEJs and ⩾5 to the other inclusion EEJ.
- For microexons: (i) ⩾10 reads mapping to the sum of exclusion EEJs or (ii) ⩾10 reads mapping to the sum of inclusion EEJs.
- For intron retention: (i) ⩾10 reads mapping to the sum of skipping EEJs or (ii) ⩾10 reads mapping to one of the two inclusion EIJs and ⩾5 to the other inclusion EIJ.
- For alternative 3′ and 5′ splice sites: ⩾10 reads mapping to the sum of all EEJs involved in the specific event.

Additional filtering was used to remove intron retention events with a binomial *P* value score above 0.05 in any of the four samples^57^.

This resulted in 365 differentially regulated AS events (Supplementary Fig. 4 and Supplementary Table 1), which were subdivided according to the predicted impact of *Nova2* knockdown on the coding sequence (Supplementary Fig. 5A): (i) AS events predicted to generate protein isoforms both when Nova2 is present or depleted (150, 41.1%); (ii) AS events predicted to trigger nonsense-mediated decay (NMD) or create a truncated protein when Nova2 is present (135, 37.0%); (iii) AS events predicted to trigger NMD or to create a truncated protein when Nova2 is absent (33, 9.0%); (iv) AS events in noncoding regions (40, 11.0%); and (v) AS events not able to be categorized into previous categories (7, 2%).

GO analysis was performed for each of the subdivided groups using ClueGO^58^. The background reference was based on multiexonic genes with the same minimum read coverage in the endothelium used above. Two categories had significant enrichment (*P* value<0.05), those generating protein isoforms ((i) 49 terms enriched in 150 genes; Supplementary Fig. 5B) and those predicted to trigger NMD/disrupted proteins when Nova2 is included ((ii) 15 terms enriched in 135 genes; Supplementary Fig. 5C). Detail of *P* values (as calculated by ClueGO) are found in Supplementary Table 2.

To compare AS events that show differential inclusion on *Nova2* knockdown in ECs to those regulated by Nova proteins in neural cells, we used the 325 cassette exons previously predicted to be Nova targets in the brain^33^ (Supplementary Table 3). Nearly all (319/325) exons could be matched to *vast-tools* AS event IDs based on the exact splice site coordinates and official gene symbols. Of these, 195/319 (61%) had enough read coverage to confidently derive inclusion estimates (PSIs) in all four EC RNAseq samples. From these, 28/195 (14.4%) exons showed a ΔPSI⩾10 on *Nova2* knockdown in ECs in the same direction as previously predicted for Nova proteins in the brain, compared with only 2/195 (1%) in the opposite direction (Supplementary Fig. 6). This overlap between Nova-regulated exons in endothelial and neural cells is highly significant (*P*=1.93*e*^−11^, hypergeometric test; background event set corresponded to 14,970 cassette exons with a minimal read coverage in the endothelium as described above and in genes with a cRPKM>2 in neurons^19^). Moreover, several methodological differences suggest that the actual overlapping of Nova2 regulation between endothelial and neural cells may be even higher. First, predictions of Zhang *et al*.^33^ include Nova1 and Nova2 targets, which may not be fully redundant. Second, exons described in Zhang *et al*. are predicted to be Nova protein targets based on Nova binding and presence of binding sites, among others. However, only half of the full set of Nova targets shows measurable differences in PSI in Nova knockout mice^33^. Finally, Nova2 proteins may use different cofactors that may be differentially expressed between endothelial and neural cells.

## Additional information

**How to cite this article:** Giampietro, C. *et al*. The alternative splicing factor Nova2 regulates vascular development and lumen formation. *Nat. Commun.* 6:8479 doi: 10.1038/ncomms9479 (2015).

## Supplementary Material

Supplementary Figures, Tables, Methods and References Supplementary Figures 1-11, Supplementary Tables 4-11, Supplementary Methods and Supplementary References

Supplementary Table 1 Supplementary Table 1 contains the 365 AS events affected by Nova2 depletion in ECs.

Supplementary Table 2 Supplementary Table 2 contains the Gene Ontology (GO) categories for Nova2 target genes in ECs.

Supplementary Table 3 Supplementary Table 3 contains the comparison of differentially included cassette exons in genes expressed in both ECs (this study) and neural cells (Zhang C. et al. 2010).

Supplementary Movie 1 One 22 hpf Tg(fli1a:EGFP)y1 embryo injected with ctr-MO was time lapsed for 15 hours. The image stack is 60 micron thick, with 5 micron voxel-depth, and 10 min. delay between one stack and the next one. The embryo's head is to the left, dorsal is to the top. Intersomitic vessels develop from the dorsal aorta, ventrally located, and extend dorsally; when they approach the dorsolateral roof of the neural tube, they divide in two branches to fuse together with branches of the adjacent vessel to form the dorsal longitudinal anastomotic vessel (DLAV). During the intersomitic vessels extension, numerous filopodia extend and retract in all directions, exploring all around the vessel but in particular near the dorsal-most leading extension.

Supplementary Movie 2 One Tg(fli1a:EGFP)y1 embryo was injected at one-two cell stage with nova2 morpholino and left to grow until 22 hpf, when it was time lapsed for 15 hours. The image stack is 60 micron thick, with 5 micron voxel-depth, and 10 min. delay between one stack and the next one. The embryo's head is to the left, dorsal is to the top. One of the intersomitic vessels, during the extension to form the DLAV, grows partially retracting its fillopodia and missing to reach the DLAV.

Supplementary Movie 3 One Tg(fli1a:EGFP)y1 embryo was injected at one-two cell stage with a mixture of nova2 morpholino and morpholino-resistant nova2 mRNA and left to grow until 22 hpf, when it was time lapsed for 15 hours. The image stack is 60 micron thick, with 5 micron voxel-depth, and 10 min. delay between one stack and the next one. The embryo's head is to the left, dorsal is to the top. Intersomitic vessels growth and sprouting appear similar to that observed in embryos injected with control MO.

Supplementary Movie 4 One 48 hpf wt embryo injected with ctr-MO was placed in 3% methyl-cellulose under a dissection microscope and its anterior region (head and ventral side of the yolk) was filmed for 13 seconds by an high resolution digital camera (Nikon). A beating heart normal for its shape and size is clearly visible just below to the head.

Supplementary Movie 5 One 48 hpf wt embryo injected with MO-nova2 was placed in 3% methyl-cellulose under a dissection microscope and its anterior region (head and ventral side of the yolk) was filmed for 13 seconds by an high resolution digital camera (Nikon). We observed a small pericardiac aedema but a normal heart for shape, size and beat.
