# Supplementary material for: The alternative splicing factor Nova2 regulates vascular development and lumen formation
Source: Nat Commun. 2015 Oct 8;6:8479. doi: 10.1038/ncomms9479 (PMC4633719; doi:10.1038/ncomms9479)
Supplement: Supplementary Figures, Tables, Methods and References — Supplementary Figures 1-11, Supplementary Tables 4-11, Supplementary Methods and Supplementary References [file ncomms9479-s1.pdf]

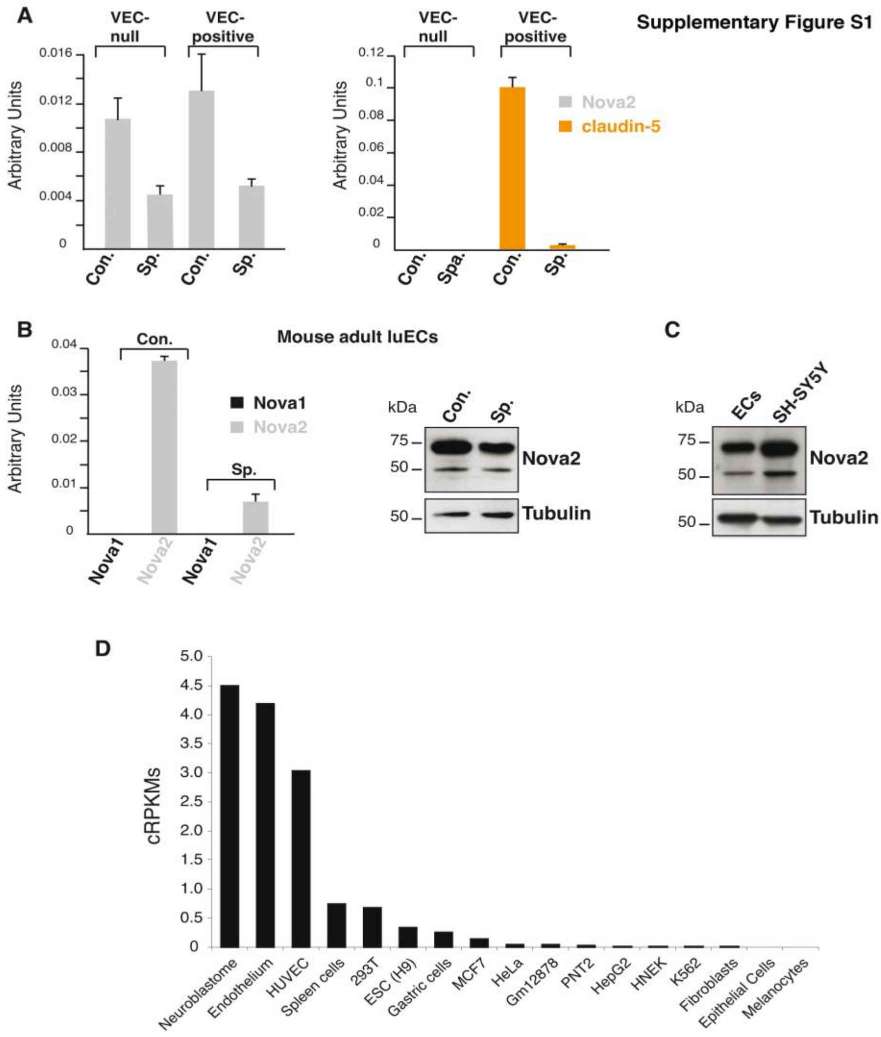

Supplementary Figure 1. Nova2 expression in different cell lines. (A) RT-qPCR analysis of *Nova2* and (as a control) *claudin-5* mRNA expression levels in confluent and sparse VEC-null and VEC-positive ECs. **(B)** RT-qPCR analysis of *Nova2* and *Nova1* mRNA expression levels (left) and immunoblotting analysis of Nova2 and Tubulin levels (right) in adult ECs from mouse lung (luECs) grown as confluent and sparse. **(C)** Immunoblotting analysis of Nova2 and Tubulin levels in mouse ECs and in human neuroblastoma SH-SY5Y cells. **(D)** *Nova2* expression levels in endothelium and different human cell lines based on RNA-seq data. cRPKMs, corrected (for mappability) reads per kilobasepair and million mapped reads. In all histograms, error bars indicate ±SD calculated from three independent experiments (n=3).

A

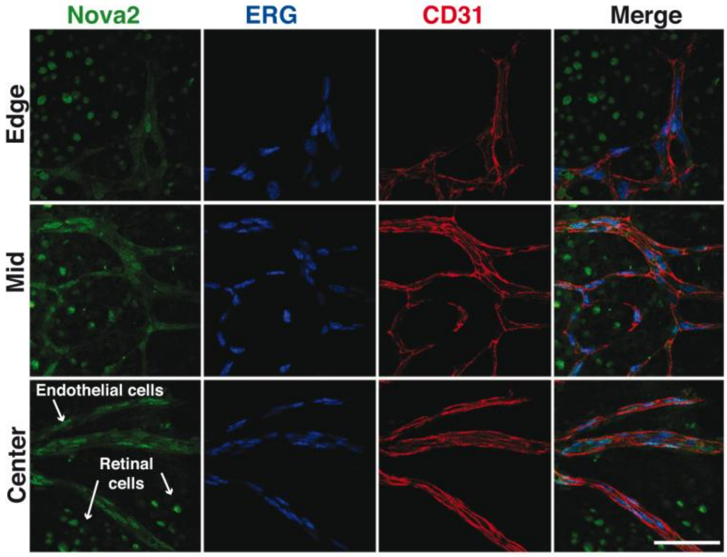

B

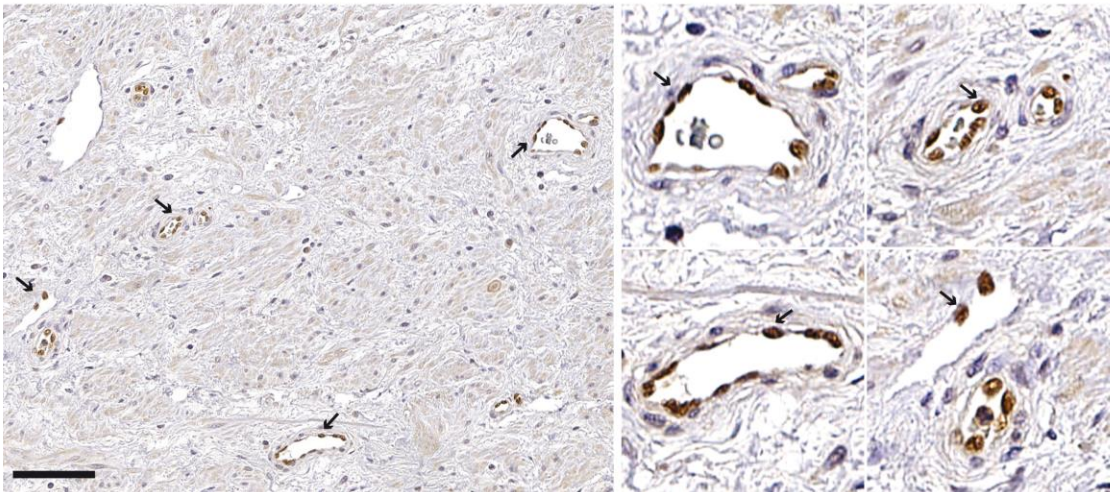

Supplementary Figure 2. Nova2 expression in the post-natal mouse retina and in normal prostate. (A) Immunofluorescence analysis of Nova2 (green), the endothelial markers CD31 (red) and ERG (blue, EC transcription factor) in whole-mounted post-natal (P6) mouse retina. Optical sections captured by confocal microscopy display large vessels in the central retina region (bottom panel), capillaries in the mid retina region (mid panel) and sprouting ECs in the leading edge of the growing vasculature (upper panel) Arrows indicate neural cells of retina and ECs of vessels expressing Nova2 (bar 50 µm). **(B)** Immunohistochemistry of Nova2 in normal human prostate (bar 100 µm); arrows indicate Nova2 nuclear staining of ECs in the blood vessels; high magnification imaging of blood vessels on the right.

Supplementary Figure S3

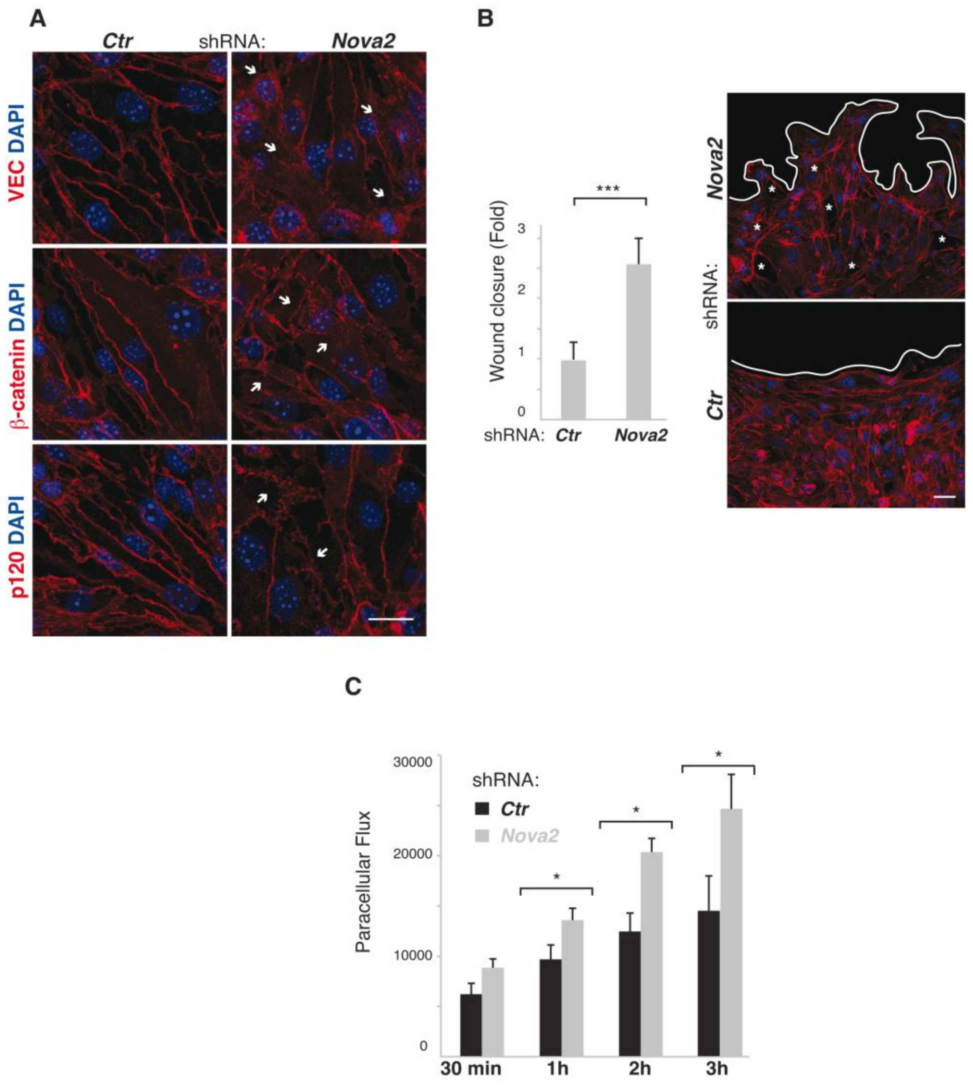

Supplementary Figure 3. Nova2 regulates adhesions, migration and permeability of ECs. (A) Immunofluorescence analysis of VEC, β-catenin and p120 (red) and DAPI (blue) in control (*Ctr*) or *Nova2* knockdown mouse ECs (bar 20 µm). **(B)** Fold increase in migration of *Nova2* knockdown mouse ECs compared with *Ctr* cells in wound healing assay (error bars ±SD; asterisks p-value < 0.001 calculated by two-tailed, unpaired, t-test n=3). On the right, staining of phalloidin for visualization of actin-containing structures (red) and DAPI (blue) 8 hours after wounding of *Ctr* or *Nova2* knockdown mouse EC monolayers (bar 20 µm). Asterisks show migrating ECs that have in part lost their contacts with neighboring cells. **(C)** Paracellular tracer flux assay. Permeability to FITC-dextran (70 kDa) was assayed. At all time values from 1h onwards, *Nova2* knockdown ECs were statistically lower than control (error bars ±SD; asterisks p-value < 0.05 calculated by two-tailed, unpaired, t-test n=3).

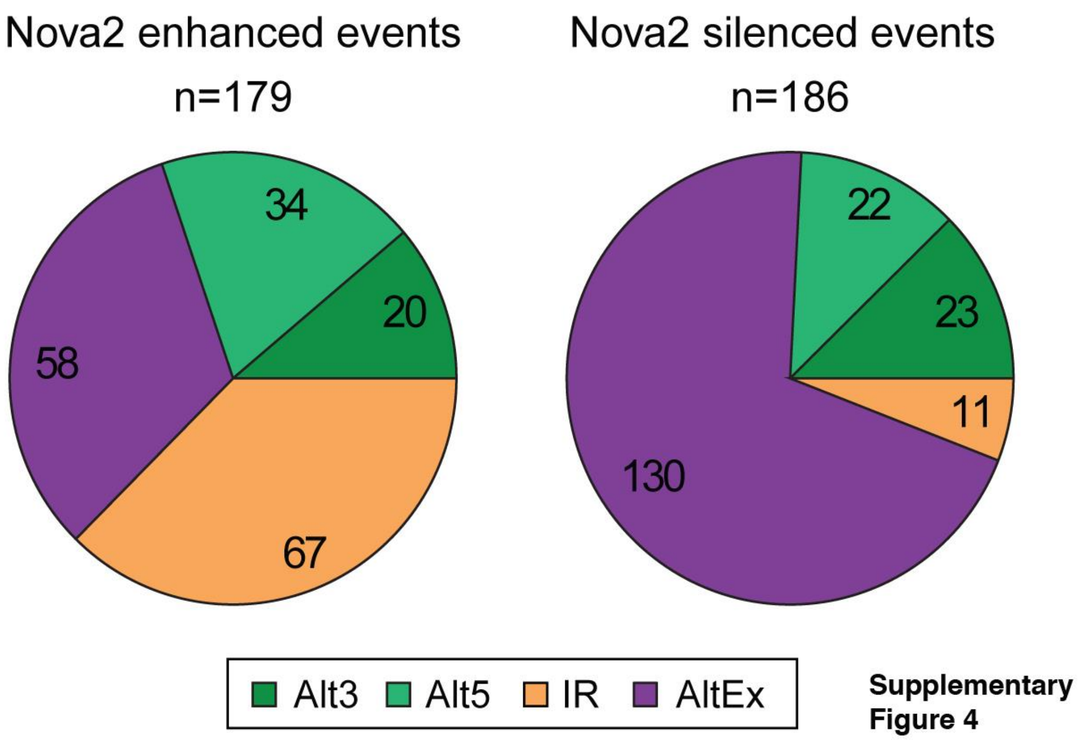

Supplementary Figure 4. Nova2 regulated AS events in ECs. By performing high-throughput RNA sequencing (RNA-Seq) of *Nova2* knockdown and control ECs, we identified 365 AS events affected by Nova2 depletion that we have classified in the major forms of alternative splicing mechanism: alternative use of an internal cassette exon (AltEx), intron retention (IR), alternative 5' splice sites (Alt5) and alternative 3' splice sites (Alt3).

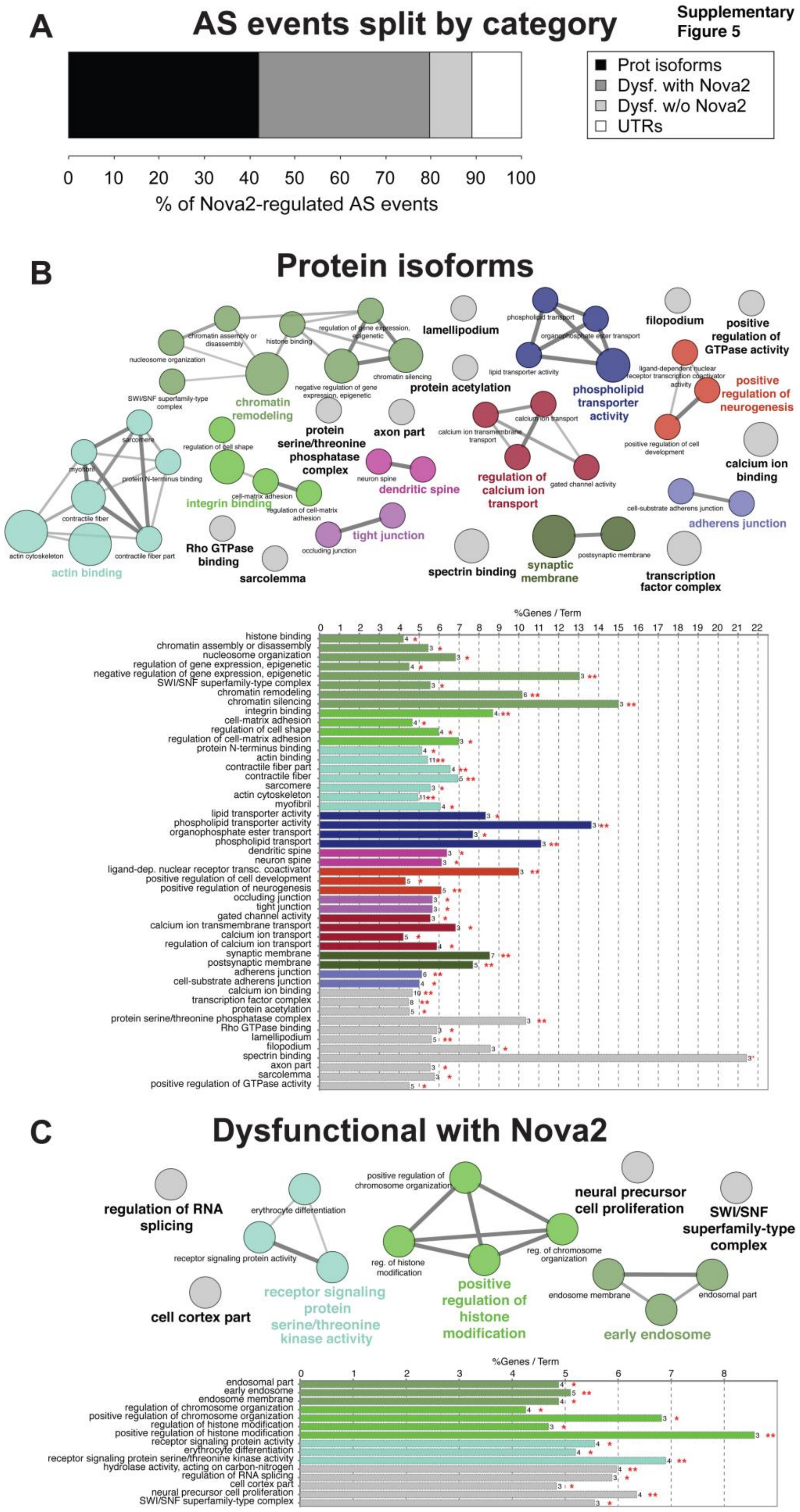

Supplementary Figure 5
A
AS events split by category
Prot isoforms
Dysf. with Nova2
Dysf. w/o Nova2
UTRs
0 10 20 30 40 50 60 70 80 90 100
% of Nova2-regulated AS events
B
Protein isoforms
chromatin remodeling
lamellipodium
phospholipid transporter activity
filopodium
positive regulation of GTPase activity
protein acetylation
positive regulation of neurogenesis
protein serine/threonine phosphatase complex
axon part
calcium ion binding
regulation of calcium ion transport
integrin binding
dendritic spine
adherens junction
tight junction
actin binding
Rho GTPase binding
sarcolemma
spectrin binding
synaptic membrane
transcription factor complex
%Genes / Term
histone binding
chromatin assembly or disassembly
nucleosome organization
regulation of gene expression, epigenetic
negative regulation of gene expression, epigenetic
SWI/SNF superfamily-type complex
chromatin remodeling
chromatin silencing
integrin binding
cell-matrix adhesion
regulation of cell shape
regulation of cell-matrix adhesion
protein N-terminus binding
actin binding
contractile fiber part
contractile fiber
sarcomere
actin cytoskeleton
myofibril
lipid transporter activity
phospholipid transporter activity
organophosphate ester transport
phospholipid transport
dendritic spine
neuron spine
ligand-dep. nuclear receptor transc. coactivator
positive regulation of cell development
positive regulation of neurogenesis
occluding junction
tight junction
gated channel activity
calcium ion transmembrane transport
calcium ion transport
regulation of calcium ion transport
synaptic membrane
postsynaptic membrane
adherens junction
cell-substrate adherens junction
calcium ion binding
transcription factor complex
protein acetylation
protein serine/threonine phosphatase complex
Rho GTPase binding
lamellipodium
filopodium
spectrin binding
axon part
sarcolemma
positive regulation of GTPase activity
C
Dysfunctional with Nova2
regulation of RNA splicing
cell cortex part
receptor signaling protein serine/threonine kinase activity
positive regulation of histone modification
neural precursor cell proliferation
SWI/SNF superfamily-type complex
early endosome
%Genes / Term
endosomal part
early endosome
endosome membrane
regulation of chromosome organization
positive regulation of chromosome organization
regulation of histone modification
positive regulation of histone modification
receptor signaling protein activity
erythrocyte differentiation
receptor signaling protein serine/threonine kinase activity
hydrolase activity, acting on carbon-nitrogen
regulation of RNA splicing
cell cortex part
neural precursor cell proliferation
SWI/SNF superfamily-type complex

Supplementary Figure 5. GO enrichment of AS events. (A) The 365 Nova2 differentially regulated AS events are split into: (i) AS events predicted to generate protein isoforms both when Nova2 is present or depleted (Prot. isoforms); (ii) AS events predicted to trigger non-sense mediated decay (NMD) or create a truncated protein when Nova2 is present (Dysf. with Nova2); (iii) AS events predicted to trigger NMD or create a truncated protein when Nova2 is absent (Dysf. w/o Nova2) and (iv) AS events in non-coding regions (UTRs). **(B)** Significantly enriched GO terms in genes containing AS events predicted to produce alternative protein isoforms ((i) from above). Top: network representation of enriched GO terms generated by ClueGO[59]. GO terms are represented as nodes based on their kappa score level (> 0.4). The node size represents the term enrichment significance. Bottom: bars represent the percentage of genes per GO term for each network above and bar labels are the exact numbers of genes associated. Significance is indicated by "**" (p-value < 0.001) or "*" (0.001 < p-value < 0.05) (p-value calculated by ClueGO). **(C)** GO enrichment analysis of events predicted to trigger non-sense mediated decay (NMD) or create a truncated protein when Nova2 is present (ii). Top/Bottom: Same representations as in "B".

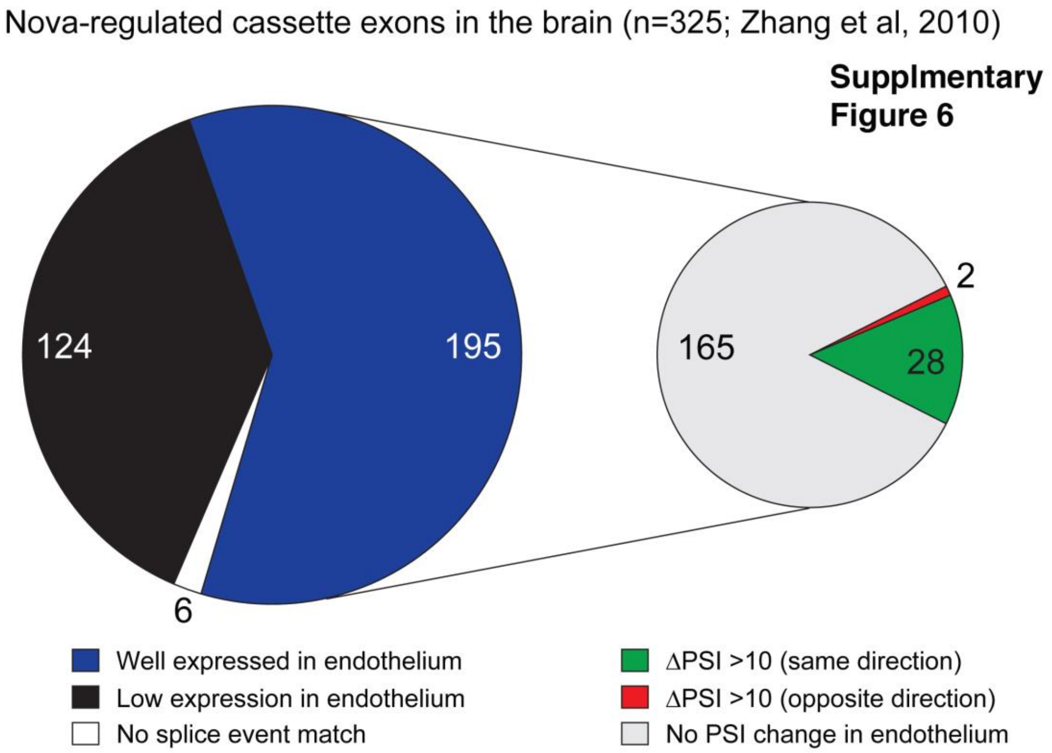

Supplementary Figure 6. Comparison between Nova-regulated cassette exons in neural and endothelial cells. Left: of the 325 predicted Nova-regulated cassette exons in[33], 195 exons have enough read coverage to confidently estimate PSI in our samples compared to 124 that do not; a further 6 exons could not be matched to our data. Right: out of the 195 exons with enough read coverage, 28 show a change in PSI>10 upon Nova2 knockdown in ECs in the expected direction, 2 in the opposite, and 165 did not show a significant PSI change.

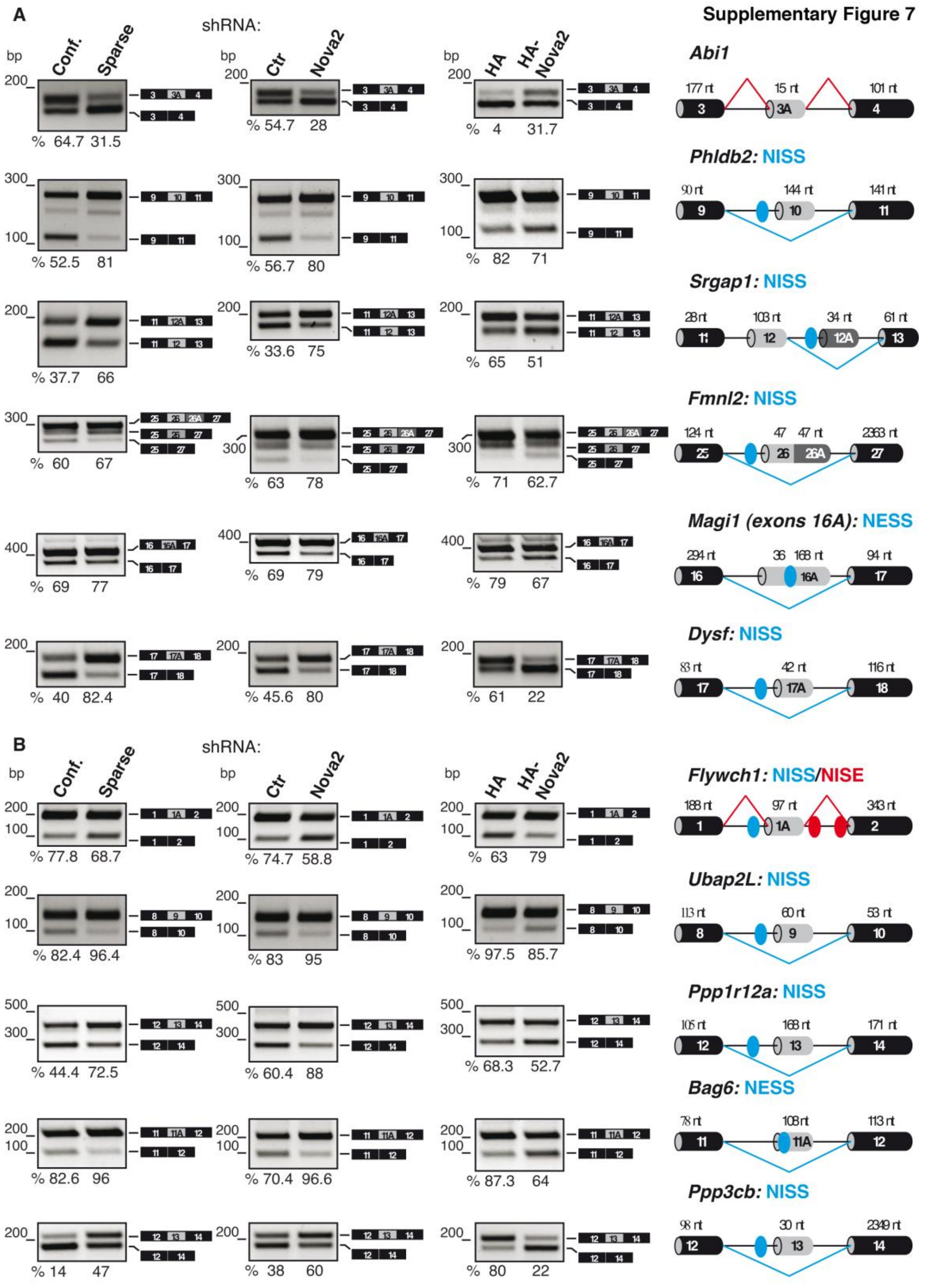

Supplementary Figure 7. Validation of novel Nova2 target exons in ECs. (A) RT-PCR analysis of selected new Nova2-regulated AS exons encoding for cell polarity, cell shape, motility and adhesion regulators in: confluent and sparse mouse ECs (left), in confluent *Nova2* knockdown ECs (middle) and in sparse ECs overexpressing HA-

tagged Nova2 (right). **(B)** RT-PCR validation of additional novel Nova2-regulated AS in the same samples as in panel A. With *Dock9* showed in Fig. 4, 12/12 (100%) novel Nova2-regulated events were validated by RT-PCR. 11/12 (91,6%) of these events (*Dock9*, *Phldb2*, *Srgap1*, *Fmnl2*, *Magi1*-exon16A, *Dysf*, *Flywch1*, *Ubap2L*, *Ppp1r12a*, *Bag6*, and *Ppp3cb*) are associated with YCAY clusters located in positions consistent with Nova previously defined mode of regulation[21, 60], suggesting they are direct Nova2 targets. The schematic representations of the genomic region containing the AS exon, the transcripts generated from skipping/inclusion of the AS exon and the calculated percentage of exon inclusion are indicated. For *Fmnl2* the percentage indicates the ratio between the isoform containing exons 26 plus 26A and total. Gray boxes, AS exons; black boxes, constitutive exons; blue/red dots indicate YCAY clusters predicted to function as Nova silencer/enhancer. Blue/red bars indicate Nova silenced/enhanced exon inclusion. NISS, Nova intronic splicing silencer; NISE, Nova intronic splicing enhancer; NESS, Nova exonic splicing silencer.

Supplementary Figure 8

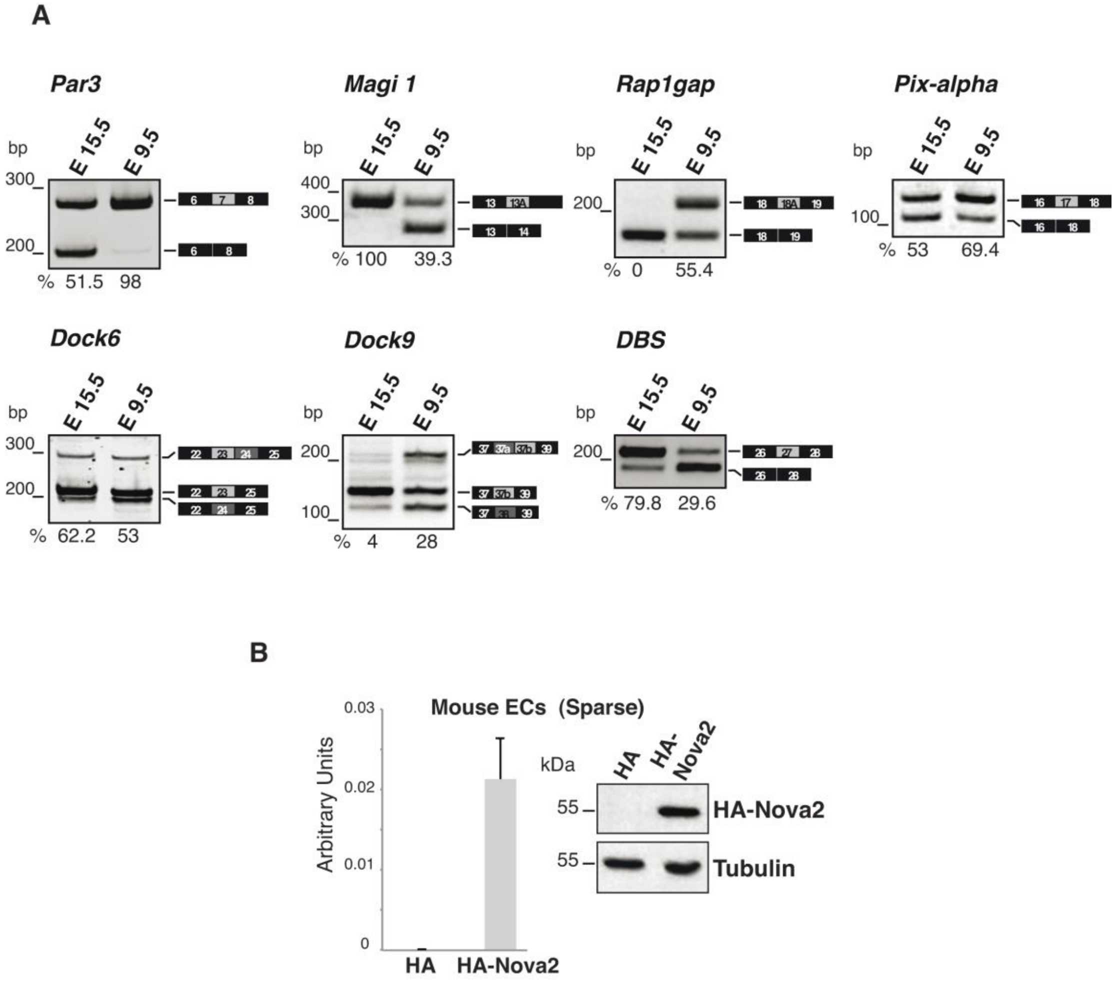

Supplementary Figure 8. Nova2 overexpressing ECs and AS profile of Nova2 targets in mouse brain. (A) AS of the indicated Nova2 pre-mRNA targets as determined by RT-PCR in E9.5 and E15.5 mouse whole brain. For each gene, the transcripts generated from skipping or inclusion of the AS exon and the calculated percentage of exon inclusion are indicated. For *Dock6* the percentage indicates the ratio between the isoform containing exon 23 (skipping exon 24) and total, whereas for *Dock9* the percentage is the ratio between the isoform containing exon 37a plus 37b and total. **(B)** *Nova2* mRNA and HA-tagged Nova2 protein levels in overexpressing ECs (grown as sparse). Error bars indicate mean ± SD calculated from three independent experiments (n=3). Nova2 protein level was analyzed by immunoblotting using anti-HA and Tubulin as loading control.

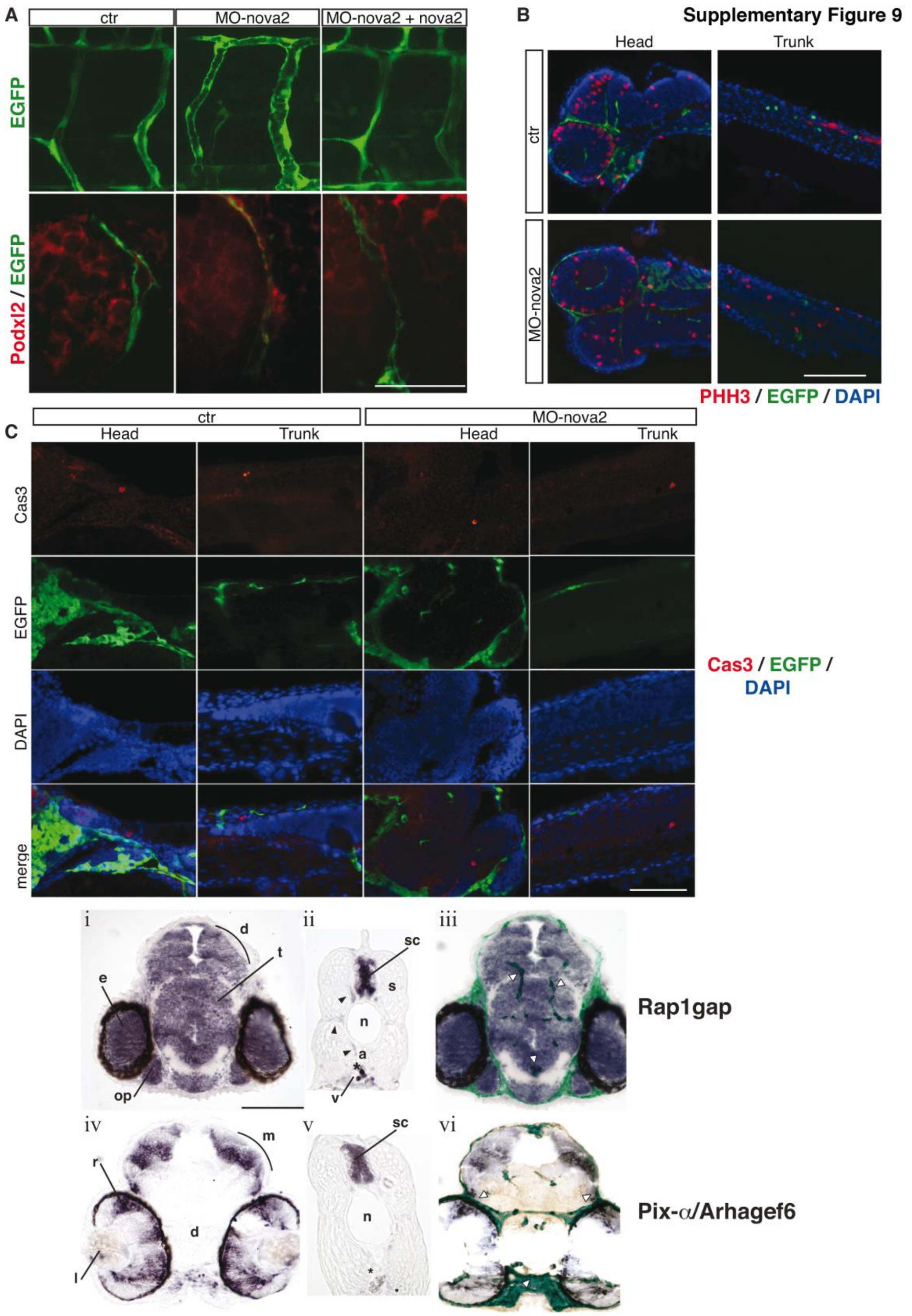

Supplementary Figure 9. Lumen, polarity, proliferation and apoptosis rate upon *nova*2 knockdown in zebrafish and expression levels of two nova2 mRNA targets in zebrafish. (A) Visualization of the lumen in the intersomitic vessels of *Tg(fli1a:EGFP)y1* 48 hpf embryos, expressing the enhanced green fluorescent protein

(EGFP) under the control of the endothelial-specific promoter *fli1a*, injected at one- or two-cells stage with control (*ctr*) or morpholino against *nova2* (MO-nova2) and in the complemented embryos (MO-nova2+nova2). Lower panel: *Tg(fli1a:EGFP)y1* 48 hpf zebrafish embryos were treated with control (*ctr*) or *nova2* morpholino (MO-nova2) oligos, fixed with PFA 4% in PBS and included in paraffin. 10 μm microtome sections of their trunk were then analyzed with anti-GFP (green) and anti-Podocalyxin (Podxl2; red) antibodies at the level of the intersomitic vessels (ISVs). Co-injection of a morpholino-resistant zebrafish *nova2* mRNA rescued the altered localization of Podxl2 in *nova2* morphant (MO-nova2+nova2). **(B)** Phosphohistone H3 (PHH3, red), EGFP (green) and DAPI (blue) staining in the trunk and head of *ctr* and *nova2* morphants. **(C)** Cleaved caspase-3 (red), EGFP (green) and DAPI (blue) staining in the trunk and head of *ctr* and *nova2* morphants. Lower panel: *Rap1gap* and *Pix-α/Arhagef6* mRNA expression levels were detected by *in situ* hybridization with LNA oligonucleotides using paraffin 5 μm sections of 48 hpf zebrafish embryos: (i and iv) trasversal sections of the head; (ii and v) trasversal sections of the trunk; (iii and vi) LNA *in situ* hybridization and anti-EGFP staining on the same trasversal sections of the head. a, aorta; d, diencephalon; e, eye; l, lens; m, mesencephlaon; n, nothocord; op, olphactory placode; r, retina; s, somite; sc, spinal chord; t, telencephalon; v, vein. Black arrowheads indicate ISVs, white arrowheads indicate head vessels. Asterisk in ii) indicates labeled blood cells present in the lumen of the vein. Scale bars 50 μm.

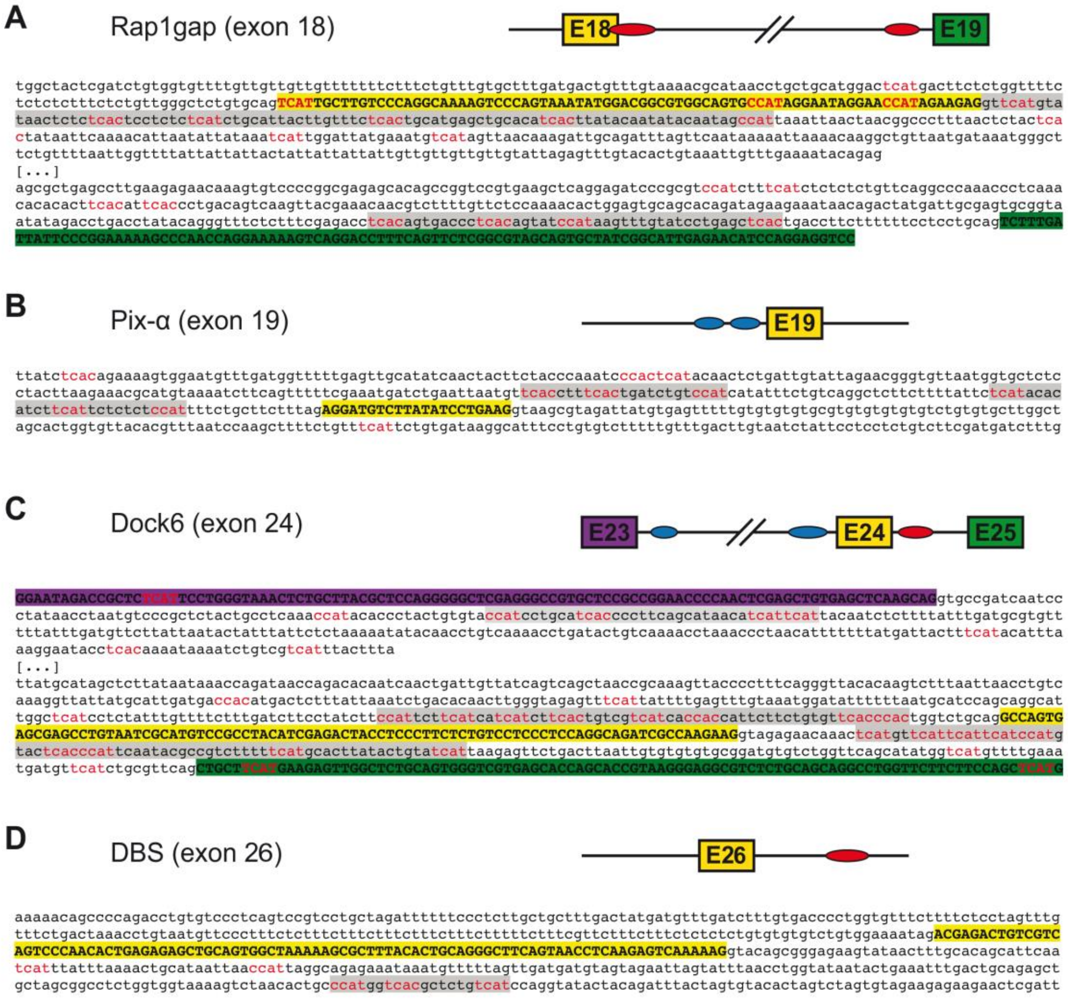

Supplementary Figure 10. Location of putative Nova binding sites correlates with the observed splicing patterns upon *nova2* knockdown in zebrafish. Nova proteins promote or repress exon inclusion depending on the locations of their binding sites (YCAY cluster elements) in pre-mRNA targets[21, 60]. In particular, Nova proteins usually promote exon skipping when bound to the exonic or upstream intronic region, while they stimulate exon inclusion when bound to downstream intronic regions.

(A) The zebrafish *Rap1gap* gene contains a large YCAY cluster (gray) in the down-stream intron that includes the 3' end of the AS exon 18 (yellow) and the 5' splice site. Another significant cluster is also present near the down-stream constitutive exon 19 (green).

(B) As in mouse, the zebrafish *Pix*-α gene presents a small NISS (Nova intronic splicing silencer) cluster (gray) in the intronic region upstream of the AS exon 19

(yellow).

(C) As in mouse, the *Dock6* zebrafish ortholog presents a well-conserved NISS upstream of exon 24 (yellow).

(D) As in mouse, the zebrafish *DBS* gene contains a small NISE (Nova intronic splicing enhancer) located 188 nt from the 3' end of the AS exon 26 (yellow).

Introns are in lowercase, exons in uppercase, and YCAY motifs are in red. Blue/red ellipses in the schemes depict predicted Nova splicing silencers/enhancers.

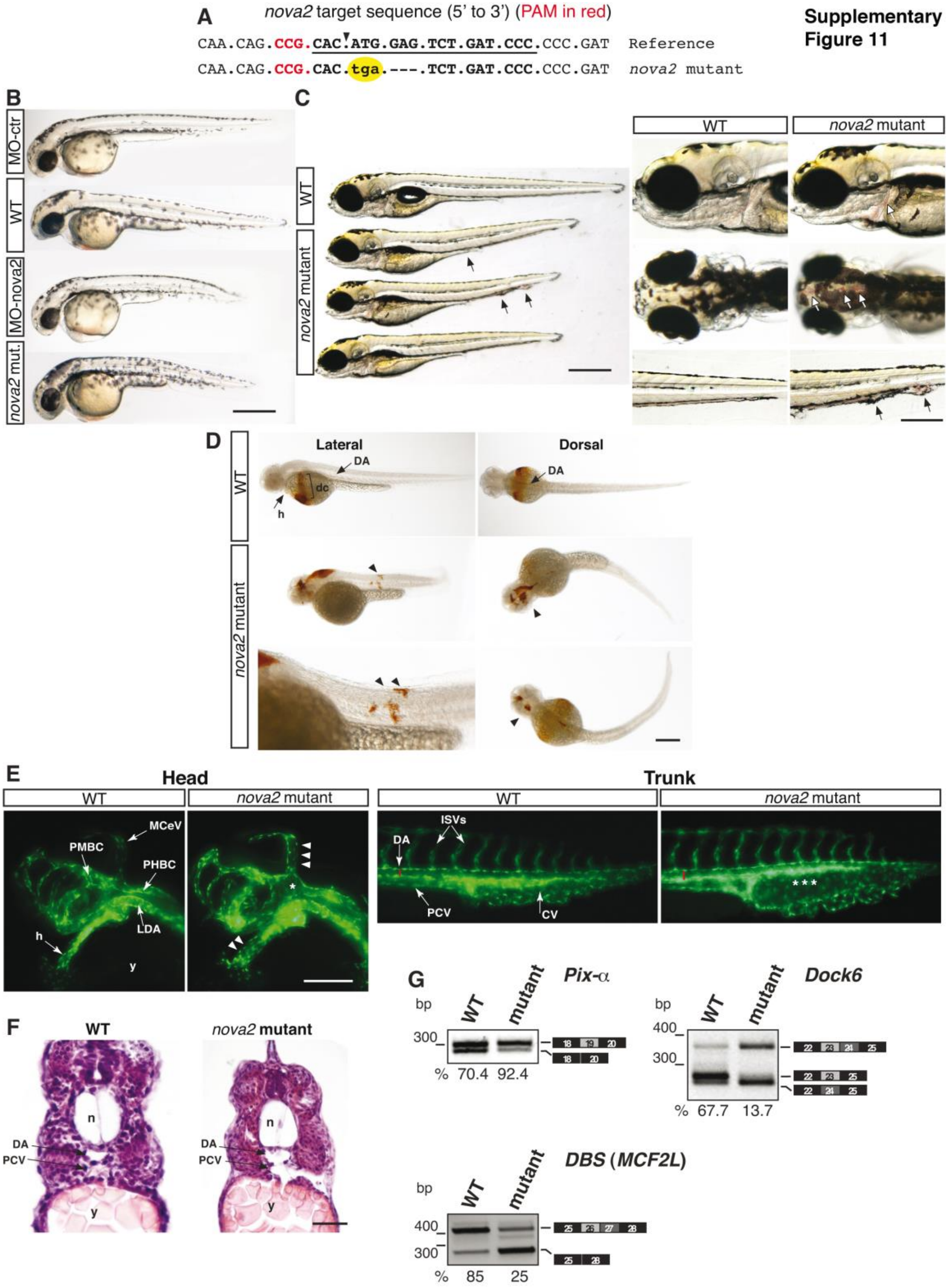

Supplementary Figure 11. Morphological, vascular and molecular defects of zebrafish *nova2* mutants parallel that observed in *nova2* morphants.

(A) Zebrafish *nova2* reference genome sequence showing the guide RNA (gRNA) target sequence (bold underlined) and the protospacer-adjacent motif (PAM, in red).

Arrowhead designates the predicted Cas9 cleavage site. Sequence of the Cas9-induced mutation is also shown (yellow oval, stop codon; –, deletion). **(B)** Lateral views of MO-control injected and wt sibling zebrafish embryos compared to MO-nova2 injected and homozygous *nova2* mutant embryos, at 2 days postfertilization (dpf) (bar 250 μm). **(C)** Lateral and dorsal views of wt sibling and homozygous *nova2* mutants at 4 dpf. Compared to siblings, *nova2* mutant embryos showed increased areas of hemorrhage in the head and trunk (black and white arrows) (bar 250 μm in panel on the left; bar 100 μm in panel on the right). **(D)** *nova2* mutants were stained at 3 dpf with o-dianisidine, which detects hemoglobin-expressing cells, in order to better visualize areas of hemorrhage (arrowheads, bar 250 μm). **(E)** Confocal analysis (lateral views) at 28 hpf showing that *nova2* mutant *Tg(kdrl:EGFP)* line (expressing the EGFP reporter in the vascular endothelium) recapitulated the altered lumen size of both cephalic and trunk blood vessels, and displayed enlarged lumen (red bar) of dorsal aorta (DA) (bar 50 μm) as observed in *nova2* morphants. Asterisks and arrowheads underline pronounced enlargements of the vascular lumen. **(F)** High magnifications of paraffin 10 μm transversal sections, stained with hematoxylin-eosin, of the trunk region of 3 days *nova2* mutant embryos highlighted alterations of the lumen size of DA and posterior cardinal vein (PCV) (bar 50 μm). **(G)** AS changes of nova2 targets at 72 hpf. *nova2* mutants showed AS changes comprable to that of *nova2* morphants (see Fig. 5e). The percentage of the inclusion of the AS exon (gray) is indicated. For *Dock6* the percentage indicates the ratio between the isoform containing exon 23 and total, whereas for *DBS* is the ratio between the isoform containing exons 26 plus 27 and total. MCeV, middle cerebral vein; PHBC, primordial hindbrain channels; PMBC, primordial midbrain channel; LDA, lateral dorsal aorta; h, hindbrain; y, yolk; n, notochord; ISVs, intersomitic vessels; dc, duct of cuvier.

Supplementary Table 1, 2 and 3, containing the RNAseq data, are in separate Excel files.

Supplementary Table 1. Information regarding the 365 AS events affected by Nova2 depletion in ECs. Full coordinate: chromosome: C1 donor, AS exon, C2 acceptor. Strand is "+" if C1 donor coordinate is smaller than C2 acceptor coordinate, and "-" otherwise. Alt3/Alt5, alternative splice site acceptor/donor selection; IR, intron retention; AltEx, cassette alternative exons (including microexons when length ≤ 27 nt). For each sample, two columns provide AS information as obtained from *vast-tools* (PSI and quality scores). Information on quality scores is available at https://github.com/vastgroup/vast-tools.

Supplementary Table 2. Gene Ontology (GO) categories for Nova2 target genes in ECs.

Supplementary Table 3. Comparison of differentially included cassette exons in genes expressed in both ECs (this study) and neural cells[33].

Supplementary Table 4. Known Nova2 regulated AS events[33] encoding for apical-basal polarity regulators.

The Par polarity complex, comprising Par3, Par6, PKCζ and the small GTPase Cdc42, promotes the establishment of apical-basal polarity in almost all metazoan cells[61]. In addition, the small GTPases Rac1 and Rap1 are important regulators of activity/localization of the Par complex[23, 25]. During this process, small GTPases are activated by GEFs (Guanine Nucleotide Exchange Factors), and repressed by GAPs (GTPase-Activating Proteins).

**Nova2 regulated exon**	**Function**	**Reference**
Par3	Par3 is a component of the Par polarity complex. Nova2-regulated exon encodes for the first PDZ domain (PDZ1) required for the interaction with Par6.	23, 62
Magi1(exon 13A)	Magi1 recruits at junctions Rap1, an important regulator of the activity/localization of Par complex.	25, 34.
Rap1GAP	Rap1 inhibitor.	35
RapGEF6/PDZ-GEF2	Activator of Rap1.	63
Pixα/ARHGEF6	Activator of Cdc42, a Par polarity complex component.	36
Dock6	Activator of Cdc42.	38
ARHGEF9	Activator of Cdc42.	64
DBS/MCF2L/ARHGEF14	Activator of Cdc42.	37
ARHGAP21	Cdc42 inhibitor.	65
ARHGAP26/GRAF	Cdc42 inhibitor.	66

Supplementary Table 5. Novel Nova2 regulated AS exons encoding for cell polarity, cell shape, motility and adhesion regulators.

Nova2 targets in ECs	Function	Reference
Abi1	Regulates actin polymerization and cytoskeletal architecture/remodeling. Essential for the formation of membrane protrusions where it specifically localizes.	67, 68
Phldb2/LL5β/Pleckstrin homology like domain family B member 2	Involved in appropriate organization of microtubule networks for maintaining cell structure and polarity.	69
Srgap1	Cdc42 inhibitor through the SLIT2-ROBO1 pathway. Nova2-regulated exon encodes part of the F-BAR domain involved in the formation of filopodia-like membrane protrusions.	70, 71
Fmnl2/Formin like 2 (also known as FRL3)	Elongation factor of actin filaments that functions as a Cdc42 effector promoting cell migration and actin polymerization at the tips of lamellipodia.	72, 73
Dock9/ Zizimin1	Activator of Cdc42.	74
Magi1 (exon16A)	The Nova2-regulated exon encodes part of the PDZ4 domain that binds the Notch ligand Dll1 (Delta-like 1).	75
Dysf	Transmembrane protein involved in membrane repair, which regulates EC adhesion and angiogenesis through polyubiquitination and degradation of platelet endothelial cellular adhesion molecule-1 (PECAM-1/CD31).	76

Supplementary Table 6. Primers used in PCR experiments.

**Primer**	**Sequence** (5'--3')
Mouse-Ank3-for	AAAGTGGGTGACCAGAGACG
Mouse-Ank3-rev	AGCCTGGTCAGAGCTGTCAT
Mouse-Par3(exon7)-for	GAAGAAGACAGCAGCCGAGT
Mouse-Par3(exon7)-rev	CTGTTTCGAAGATCTCCATCG
Mouse-Magi1(exon13A)-for	AGCCAGAATAGCTCCCAGCA
Mouse-Magi1(exon13A)-rev	GGTTCCCCTGGTTCATTTCC
Mouse-Rap1gap-for	TCACCCCCAACAACCCTGACC
Mouse-Rap1gap-rev	CTCTTCTCCTGAACCTCCTGGATG
Mouse-Pix-α-for	CTACGACCTGCACCTCCACT
Mouse-Pix-α-rev	CGTATTCTTCCTCGGATGCT
Mouse-Dock6-for	TCTGTGGACGATGAGGTGTC
Mouse-Dock6-rev	CTGCAGGACTAGCTCACGAA
Mouse-DBS-for	CCCACAAAAGGGAACACAAG
Mouse-DBS-rev	CGGGTCGCTCTTTACTTCGT
Mouse-Dock9-for	TTCCAGTACATGGGGAAGCG
Mouse-Dock9 rev	ACCTCAGTAGCGATGTTGGC
Mouse-Magi1(exon16A)-for	CAAGGCAGCCAGAACTCTCT
Mouse-Magi1(exon16A)-rev	GGTGATGGTGGCAATCTTCT
Mouse-Abi1-for	ATCGCACCCGCAAATATGGA
Mouse-Abi1-rev	TGGGAGGGTTTGTTCTCGAC
Mouse-Dysf-for	ACCGCCTCACTCACAATGAC
Mouse-Dysf-rev	AACTCTCGGGGACTGCCATA

Mouse-Phldb2-for	AGTCTCACTGGTGGGAAAGG
Mouse-Phldb2-rev	TCTTTCGAGGCAGAGTGTCA
Mouse-Srgap1-for	ATGACTTGCTGCAGAGGACT
Mouse-Srgap1-rev	AATCGGATGCAGCTTTCCAC
Mouse-Fmnl2-for	TGCGGAATTAAGAAGGCGACA
Mouse-Fmnl2-rev	TGAAGGTGGTCTCCAGTCAC
Zebra-Dock6-for	CCACATCAAGACCCAGTGC
Zebra-Dock6-rev	CCACTGCAGAGCCAACTCTT
Zebra-DBS-for	CCGATCAGATCTCCCCATTA
Zebra-DBS-rev	TTTCTTCCCTCCCTCATCCT
Zebra-Rap1gap-for	TGGGCCTCAATATCAGGAAG
Zebra-Rap1gap-rev	CTACGCCGAGAACTGAAAGG
Zebra-Pix-α-for	ATCGGACCATGGTGTTCTG
Zebra-Pix-α-rev	TGGGTCTGGGGCTTTTACTA
Mouse-Flywch1-for	GACACTCACACGAAGGATCG
Mouse-Flywch1-rev	CTCTGGCAGATGGGTCTCTC
Mouse-Ppp3cb-for	CCTAGTGGAGTGTTGGCTGG
Mouse-Ppp3cb-rev	GTATGTGCGGTGTTCAGGGA
Mouse-Ubap2l-for	GAGCCAGCCAACACTGATGA
Mouse-Ubap2l-rev	TCTGTTGCAGTCCTCCATGC
Mouse-Ppp1r12a-for	GTTCGATCAATGAAGGATCCA
Mouse-Ppp1r12a-rev	CCGTCTAGACTGTCTTGCTT
Mouse-Bag6-for	TCTGGAGCACAGCCTGGTG
Mouse-Bag6-rev	GAGTTGGTGCTGTTGGGAAG

Supplementary Table 7. Primers used in qPCR experiments.

**Primer**	**Sequence** (5'--3')
Mouse-Nova2-for	AGGACTGATCATCGGTAAGG
Mouse-Nova2-rev	GGGTCTTCCTGTACCTTCTG
Human-Nova2-for	CAGCTTTATTGCCGAGAAGG
Human-Nova2-rev	ACCCATGCTCCTGACTGTTC
Mouse-Nova1-for	GCCAGTACTTTCTAAAGGTTCTCA
Mouse-Nova1-for	AGTGGCTCCAGTTTCTTTTTG
Mouse-Human-GAPDH-for	TCAAGAAGGTGGTGAAGCAGG
Mouse-Human-GAPDH-rev	ACCAGGAAATGAGCTTGACAAA
Mouse-Ubiquitin-for	TCTTCGTGAAGACCCTGACC
Mouse-Ubiquitin-rev	CAGGTGCAGGGTTGACTCTT
Mouse-beta-actin-for	TCCACACCCGCCACCAG
Mouse-beta-actin-rev	CCATTCCCACCATCACACCCTG
Mouse-Mbnl1-for	AGAGATCTTGCCGACTGCAC
Mouse-Mbnl1-rev	AATTGCCACGCTGGTACTCT
Mouse-Mbnl2-for	GCCCAGCAGATGCAATTTAT
Mouse-Mbnl2-rev	TGGGACTAACCCAACTCCAG
Mouse-Mbnl3-for	CTCATGCTGCAGAACGCTCA
Mouse-Mbnl3-rev	GAACTCCTGGCAGTGCAAGA
Mouse-claudin5-for	GGCACTCTTTGTTACCTTGACC
Mouse-claudin5-rev	CAGCTCGTACTTCTGTGACACC
Human-hRPL32-for	AGTTCCTGGTCCACAACGTC
Human-hRPL32-rev	TGCACATGAGCTGCCTACTC

Supplementary Table 8. Morpholinos were purchased from Gene Tools (Philomath, Oregon, USA).

**oligonucleotide**	**Sequence** (5'--3')
MO-Ctr	CCTCTTACCTCAGTTACAATTTATA
MO-nova2	TGCACTGCCCCACCGGCCATCATTT

Supplementary Table 9. Primers used in cloning experiments.

**Primer**	**Sequence** (5'--3')
CG13R	TTAGCCCACCTTCTGCGGATT
CG13F	GACCGAGCCTAAAGCTTCTTT
CG30F	CCGCCATGGCTGGTGGGGCAGTGCAGCAAAACG
SG55-F	CAATCACGGATGTGGCTTAG
SG55-R	TTTGGGGCTCTTACTAGATTCCTCTTTATAACCTAGTGCTGC
SG56-F	GCAGCACTAGGTTATAAAGAGGAATCTAGTAAGAGCCCCAAA
SG56-R	GCGAATTCTTACTGGAGAATCGAGGTCTT
SG57-F	GCGGATCCACCATGTACCCATACGATGTTCCAGATTACGCTGG ACAATCACGGATGTGGCTTAG

Supplementary Table 10. Primers used for gene specific retrotranscription (GSP).

**Primer**	**Sequence** (5'--3')
Mouse-DBS-GSP	TGAGTTCCTCAGCACTGGAC
Zebra-DBS-GSP	TTTCTTCCCTCCCTCATCCT
Zebra-Pix-α-GPS	TTGAGTATCTGGGCGTCCTC

Supplementary Table 11. Primers for *nova2* gRNA production and for sequencing the *nova2* target region.

**Name**	**Sequence (5'-3')**	**Experiment**
n2 gRNA_F	TAGGGGGATCAGACTCCATGTG	gRNA syntesis
n2 gRNA_R	AAACCACATGGAGTCTGATCCC	gRNA syntesis
M13-Rev.	AACAGCTATGACCATG	pDR274 sequencing
n2-locus-F	GTGGTTTCACATTTAGAAAAC	T7EI and sequencing
n2-locus-R	CCACTGGGAGCAAAACATCAAA	T7EI and sequencing

SUPPLEMENTARY REFERENCES

60. Licatalosi, D.D., Mele, A., Fak, J.J., Ule, J., Kayikci, M., Chi, S.W., et al. HITS-CLIP yields genome-wide insights into brain alternative RNA processing. *Nature* 456: 464-469 (2008).

61. Martin-Belmonte, F. & Mostov, K. Regulation of cell polarity during epithelial morphogenesis. *Curr. Opin. Cell Biol.* **20,** 227-234 (2008).

62. Lin, D., Edwards, A.S., Fawcett, J.P., Mbamalu, G., Scott, J.D., & Pawson, T. A mammalian PAR-3-PAR-6 complex implicated in Cdc42/Rac1 and aPKC signalling and cell polarity. *Nat Cell Biol.* **2,** 540-547 (2000).

63. Dubé, N., Kooistra, M.R., Pannekoek, W.J, Vliem, M.J., Oorschot, V., Klumperman J, *et al*. The rapgef pdz-gef2 is required for maturation of cell-cell junctions. *Cell. Signal.* **20,** 1608-1615 (2008).

64. Reid, T., Bathoorn, A., Ahmadian, M.R. & Collard, J.G. Identification and characterization of hpem-2, a guanine nucleotide exchange factor specific for cdc42. *J. Biol. Chem.* **274,** 33587-33593 (1999).

65. Barcellos, K.S., Bigarella, C.L., Wagner, M.V., Vieira, K.P., Lazarini, M., Langford, P.R., *et al*. Arhgap21 protein, a new partner of α-tubulin involved in cell-

cell adhesion formation and essential for epithelial-mesenchymal transition. *J. Biol. Chem.* **288,** 2179-2189 (2013).
66. Doherty, G.J., Åhlund, M.K., Howes, M.T., Morén, B., Parton, R.G., McMahon, H.T., Lundmark, R. The endocytic protein graf1 is directed to cell-matrix adhesion sites and regulates cell spreading. *Mol. Biol. Cell* **22,** 4380-4389 (2011).
67. Roffers-Agarwal, J., Xanthos, J.B. & Miller, J.R. Regulation of actin cytoskeleton architecture by Eps8 and Abi1. BMC *Cell Biol.* **6,** 36 (2005).
68. Innocenti, M., Gerboth, S., Rottner, K., Lai, F.P., Hertzog, M., Stradal, T.E., Frittoli, E., Didry, D., Polo, S., Disanza, A., Benesch, S., Di Fiore, P.P., Carlier, M.F. & Scita, G. Abi1 regulates the activity of N-WASP and WAVE in distinct actin-based processes. *Nat Cell Biol.* **7,** 969-976 (2005).
69. Hotta, A., Kawakatsu, T., Nakatani, T., Sato, T., Matsui, C., Sukezane, T., *et al.* Laminin-based cell adhesion anchors microtubule plus ends to the epithelial cell basal cortex through LL5alpha/beta. *J Cell Biol.* **189,** 901-917 (2010).
70. Wong, K., Ren, X.R., Huang, Y.Z., Xie, Y., Liu, G., Saito, H., *et al.* Signal transduction in neuronal migration: roles of GTPase activating proteins and the small GTPase Cdc42 in the Slit-Robo pathway. *Cell.* **107,** 209-221 (2001).
71. Coutinho-Budd, J., Ghukasyan, V., Zylka, M.J. & Polleux, F. The F-BAR domains from srGAP1, srGAP2 and srGAP3 regulate membrane deformation differently. *J Cell Sci.* **125,** 3390-3401 (2012).
72. Block, J., Breitsprecher, D., Kühn, S., Winterhoff, M., Kage, F., Geffers, R., *et al.* FMNL2 drives actin-based protrusion and migration downstream of Cdc42. Curr Biol. **22,** 1005-1012 (2012).
73. Kühn, S. & Geyer, M. Formins as effector proteins of Rho GTPases. *Small GTPases.* 5, e29513 (2014).
74. Gadea, G. & Blangy, A. Dock-family exchange factors in cell migration and disease. *Eur J Cell Biol.* **93,** 466-477 (2014).
75. Mizuhara, E., Nakatani, T., Minaki, Y., Sakamoto, Y., Ono, Y., Takai Y. MAGI1 recruits Dll1 to cadherin-based adherens junctions and stabilizes it on the cell surface. *J Biol Chem.* **280**, 26499-26507 (2005).
76. Sharma, A., Yu, C., Leung, C., Trane, A., Lau, M., Utokaparch, S., *et al.* A new role for the muscle repair protein dysferlin in endothelial cell adhesion and angiogenesis. *Arterioscler Thromb Vasc Biol.* **30,** 2196-2204 (2010).
